# Synergistic Effect of Dual‐Functional Groups in MOF‐Modified Separators for Efficient Lithium‐Ion Transport and Polysulfide Management of Lithium‐Sulfur Batteries

**DOI:** 10.1002/advs.202515034

**Published:** 2025-09-19

**Authors:** Zheng Liu, Wanchang Feng, Haoyang Xu, Zilin Yang, Wenting Li, Mohsen Shakouri, Hsiao‐Chien Chen, Fan Zhang, Huan Pang

**Affiliations:** ^1^ School of Chemistry and Chemical Engineering Yangzhou University Yangzhou Jiangsu 225002 P. R. China; ^2^ Key Laboratory of Advanced Energy Materials Chemistry (Ministry of Education) Nankai University Tianjin 300071 P. R. China; ^3^ Contemporary Amperex Technology Co., Ltd Ningde 352100 P. R. China; ^4^ Canadian Light Source Inc. University of Saskatchewan Saskatoon S7N 2V3 Canada; ^5^ Center for Reliability Science and Technologies Chang Gung University Kidney Research Center Department of Nephrology Chang Gung Memorial Hospital Taoyuan 33302 P. R. China

**Keywords:** energy storage, functional groups, lithium‐ion transport, lithium‐sulfur batteries, metal‐organic frameworks

## Abstract

Lithium‐sulfur batteries (LSBs) have been regarded as an attractive candidate for future energy storage systems owing to their exceptionally high energy density. However, the further application of LSBs is faced with critical challenges such as the intrinsic insulation of sulfur and the shuttle effect of soluble lithium polysulfides (LiPS). To overcome these problems, a dual functional metal‐organic framework (UIO‐66‐NH_2_‐HSO_3_) modified separator is proposed, strategically implemented to examine the dual functionality in anchoring LiPS and facilitating Li^+^ transport. Theoretical calculations indicated that the Li^+^ diffusion kinetics and LiPS adsorption ability are synergistically boosted by the dual‐functional groups in the framework. The ‐HSO_3_ demonstrates high affinity for capturing LiPS species while simultaneously repulsing polysulfide anions. Conversely, ‐NH_2_ effectively immobilizes these anionic species. Additionally, the lower LUMO energy level of NH_2_‐H_2_BDC and the higher HOMO energy level of HSO_3_‐H_2_BDC significantly accelerate the reaction kinetics of LSBs. Electrochemical assessments revealed that the UIO‐66‐NH_2_‐HSO_3_@PP composite delivers ultra‐high rate capability and long‐term cycling durability, surpassing most of the reported results. In situ spectroscopic analysis established that the UIO‐66‐NH_2_‐HSO_3_@PP facilitates homogeneous lithium‐ion migration while mitigating polysulfide shuttling. This study provides a theoretical foundation for the rational design of multifunctional MOFs membranes as advanced separators for high‐performance LSBs.

## Graphical abstract

This study developed a bifunctional metal‐organic framework (UIO‐66‐NH_2_‐HSO_3_) modified separator, which shows dual functionality in anchoring LiPS and facilitating Li^+^ transport. Due to the systematic modification, the assembled LSBs deliver excellent high‐rate performance and ultra‐long cycling stability. In addition, the inner mechanism of enhanced reaction kinetics of LiPS intermediates and inhibition of polysulfide shuttling effect are verified by the combination of DFT calculations and a series of in situ characterizations.

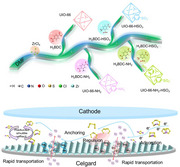

## 1 Introduction

Against the backdrop of a severe energy crisis and increasing environmental pressure, the search for sustainable clean energy solutions has become the consensus and urgent pursuit of governments, research institutes, and enterprises.^[^ ^1^, ^2^ ^]^ In this context, the progress of battery technology is particularly critical. Lithium‐ion batteries (LIBs) have become one of the most mainstream battery technologies due to their high energy density, long cycle lifetime, and low self‐discharge efficiency.^[^ ^3^, ^4^ ^]^ Nevertheless, with the continuous improvement of battery performance requirements, LIBs are still facing many challenges in terms of energy density, cost, and safety. As a consequence, researchers have begun to explore new battery technologies to meet higher energy demands and environmental standards in the future. Within the landscape of emerging battery systems, lithium‐sulfur batteries (LSBs) technology stands out as a research hotbed due to its exceptional theoretical specific energy approaching 2600 Wh kg.^−1[^ ^5^, ^6^ ^]^ Compared to traditional LIBs, LSBs not only provide higher energy storage, but also lower material costs and better environmental friendliness.^[^ ^6^, ^7^ ^]^

Despite the superior advantages of LSBs as an emerging novel energy storage technology with high‐energy‐density, they still face a series of challenges in the development of electrochemical performance.^[^ ^8^, ^9^ ^]^ During the charging and discharging process, the lithium sulfides (Li_2_S, Li_2_S_2_, etc.) are inevitably formed, derived from the reaction between sulfur and lithium, and the corresponding intermediates are resistant to complete reduction during battery cycling, leading to the loss of active material and poor cycling stability of the battery. This is primarily due to the polysulfide shuttle effect, where soluble long‐chain lithium polysulfides (LiPS) migrate between the cathode and anode, causing continuous active material loss, low Coulombic efficiency, and rapid capacity decay.^[^ ^10^, ^11^ ^]^ In addition, the poor electrochemical conductivity of sulfur can affect the overall conductivity of LSBs, resulting in a fatal rate performance. Although the conductivity can be improved by compounding with a conductive agent (carbon material), it is still difficult to satisfy the demand for high multiplication discharge. Regrettably, polysulfide migration‐induced shuttle phenomena and sluggish LiPS redox conversion kinetics remain persistent barriers impeding LSB commercialization.^[^ ^12^, ^13^, ^14^ ^]^ Various inorganic materials, including oxides, sulfides, and phosphides, have been extensively explored to tackle these issues. Conductive oxides can enhance the overall conductivity of the sulfur and battery, while other compounds can serve as sulfur carriers, effectively restricting the dissolution of LiPS and reducing the loss of active materials. Additionally, modified inorganic materials have been utilized in the development of advanced separators, which can effectively block LiPS migration while maintaining Li^+^ permeability, thereby enhancing both safety and performance.^[^ ^15^, ^16^, ^17^ ^]^ Despite the ability of inorganic materials to limit the dissolution of LIPS, it is still not possible to completely avoid the formation of LIPS and inhomogeneous mass transport, which requires further optimization of material design and battery structure.^[^ ^18^, ^19^ ^]^

Metal‐organic frameworks (MOFs) present compelling advantages for lithium‐sulfur battery systems, leveraging distinctive structural characteristics and tailorable chemistry. MOFs possess an extremely high specific surface area, which can effectively increase the contact area of active materials, thereby enhancing the energy and power densities of the batteries.^[^ ^20^, ^21^, ^22^, ^23^ ^]^ The structural stability, chemical environment, and porous structure of MOFs can be precisely modified via the rational matching between metal ions and organic ligands, enabling optimization of Li^+^ and lithium LiPS transport channels and promoting rapid ion migration.^[^ ^24^, ^25^ ^]^ Furthermore, organic linkers within MOF architectures can be tailored to introduce electroactive moieties that form favorable interactions with lithium species, thereby enhancing electrochemical behavior.^[^ ^26^, ^27^ ^]^ For example, Du's team introduced ferrocene to prepare NH_2_‐UIO‐66‐Fc functionalized separators, which inhibit polysulfide shuttling by reducing pore size and leveraging cation‐π interactions, significantly enhancing the cycling stability of LSBs.^[^ ^28^, ^29^ ^]^ However, traditional design approaches often modify MOFs with only one kind of functional group, leading to limited effectiveness in LSBs. Single‐functional‐group of MOFs are typically optimized for either Li^+^ transport or LiPS interaction but fail to address the synergistic effect between the two, leading to poor coordination between Li^+^ migration and LiPS conversion during the charging and discharging process, negatively impacting energy density and cycling stability.^[^ ^30^, ^31^ ^]^ Moreover, the complex redox reactions in LSBs involve multiple intermediates, and the single‐functional‐group of MOFs is struggled to capture and stabilize, leading to unclear reaction mechanisms. Additionally, the deep interaction mechanisms between functional groups and complex sulfide conversion products remain poorly understood. Therefore, the rational design of multifunctional MOFs to elucidate the structure‐property relationships and advance their practical application of LSBs remains a significant challenge.^[^ ^32^, ^33^ ^]^

Herein, we systematically engineered a multifunctional separator featuring ‐NH_2_ and ‐HSO_3_ moieties to circumvent the inherent constraints of monofunctional MOF materials in LSBs. DFT calculations on MOF ligands revealed that HSO_3_‐H_2_BDC exhibits high HOMO energy, while NH_2_‐H_2_BDC shows low LUMO energy, indicating that UIO‐66‐NH_2_‐HSO_3_ would demonstrate excellent reaction kinetics. Further DFT calculations on MOF ligands and clusters confirmed that the ‐SO_3_H functional groups demonstrate a strong capacity to adsorb LiPS molecules, electrostatically repel polysulfide anions, and facilitate Li^+^ transport. Conversely, the ‐NH_2_ functional groups were shown to strongly anchor polysulfide anions and significantly enhance Li^+^ migration via coordination interactions, thus guaranteeing uniform Li^+^ passage. By integrating ‐SO_3_H and ‐NH_2_ within the same MOF structure, we fabricated a functional separator modified with sulfonic acid/amine dual functional groups (UIO‐66‐NH_2_‐HSO_3_@PP). This design achieves synergistic effects combining adsorption and repulsion, enabling better control over LSPS behavior in the electrolyte, obviously improving the performance of LSBs. The LSBs assembled with the modified separator show a high initial discharge capacity of 1215.7 mAh g^−1^ at 0.5C. More significantly, under 2C cycling conditions, these modified cells revealed merely 0.085% capacity fade per cycle. After 500 cycles, it still maintains a stable capacity of 533.4 mA h g^−1^. These results demonstrate the superior cycling stability and high‐capacity performance of UIO‐66‐NH_2_‐HSO_3_@PP. This investigation provides novel insights for exploring MOFs with multiple functional groups as LSBs separators in accelerating reaction kinetics and inhibiting LIPS shuttling.

## 2 Results and Discussion

**Figure 1:**
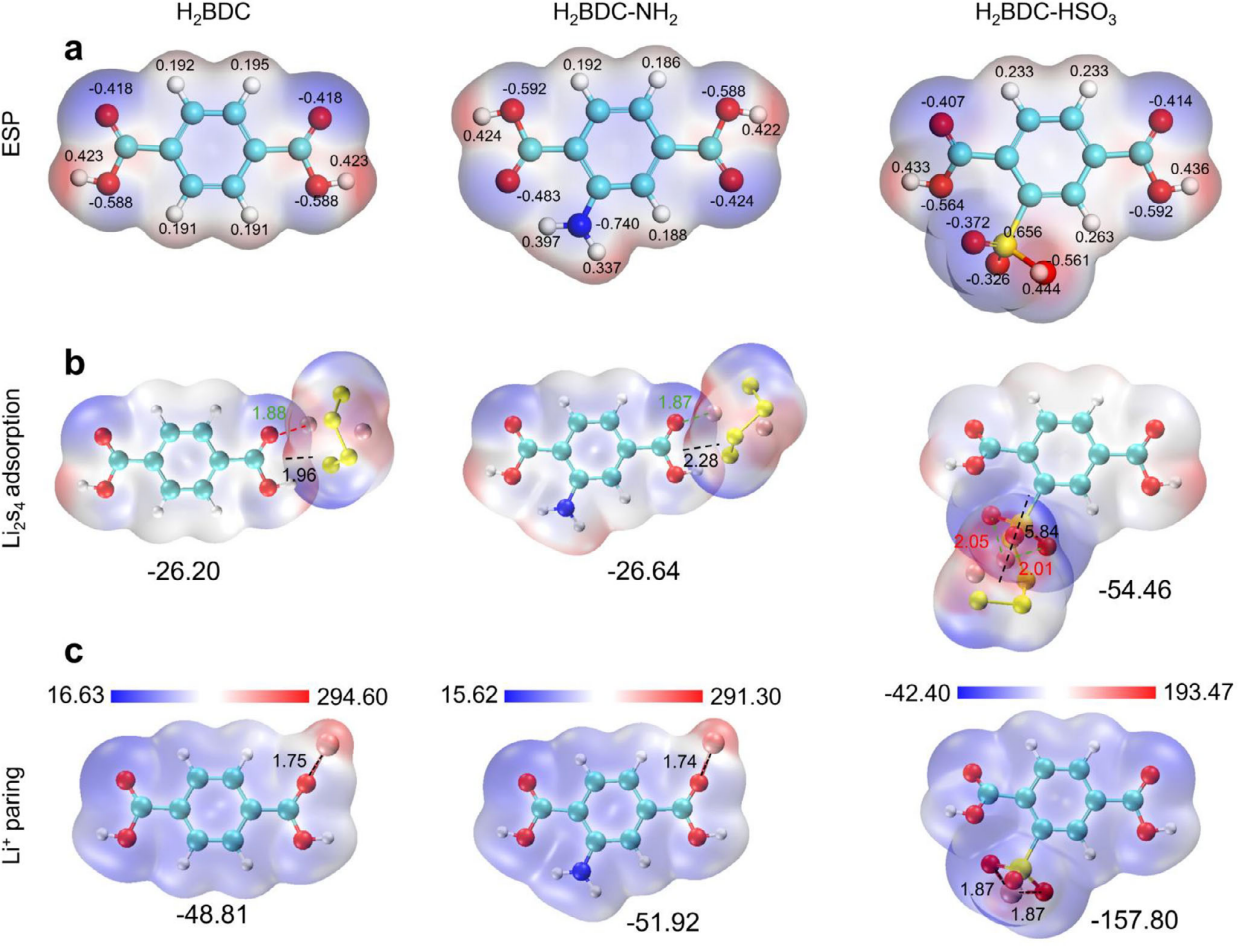
a) ESP Maps, b) Chemical adsorption configuration of Li_2_S_4,_ and c) Lithium ions pairing configuration of H_2_BDC, NH_2_‐H_2_BDC, and HSO_3_‐H_2_BDC.

To elucidate the intrinsic effect mechanism of different functional moieties on the chemo physical properties of MOFs, the density functional theoretical (DFT) calculations on ligands bearing varied substituents are initially carried out. **Figure** **1**a shows the electrostatic potential distributions (ESP) of H_2_BDC, NH_2_‐H_2_BDC, and HSO_3_‐H_2_BDC.^[^ ^34^ ^]^ The highest and lowest potentials of H_2_BDC and NH_2_‐H_2_BDC are +52.35 kcal mol^−1^, ‐30.29 kcal mol^−1^ and +52.94 kcal mol^−1^, ‐32.88 kcal mol^−1^. The increased potential can be attributed to the modulation of electron distribution within the benzene ring through conjugated and inductive pathways with a strongly electron‐donating ‐NH_2_ moiety. The lone pair of electrons on ‐NH_2_ conjugates with the π‐electron system of the benzene ring, increasing the electron cloud density. Additionally, the high electronegativity of the nitrogen atom in ‐NH_2_ attracts electrons on the benzene ring through an inductive effect, further increasing the electron density near the amino group. This enhanced electron density of NH_2_‐H_2_BDC can effectively increase the electrochemical activity of MOFs. In contrast, ‐SO_3_H, as a strong electron‐withdrawing group, significantly alters the electron cloud density of HSO_3_‐H_2_BDC by affecting its molecular structure and electron distribution, with its highest and lowest potentials being +0.11 and ‐125.77 kcal mol^−1^, respectively. Li_2_S_4_ was selected as a representative of LiPS to evaluate the adsorption behavior. Figure 1b reveals the binding capability of H_2_BDC, NH_2_‐H_2_BDC, and HSO_3_‐H_2_BDC substrates toward Li_2_S_4_. The binding energy for Li_2_S_4_ on sulfonic acid‐functionalized H_2_BDC (‐54.46 kcal mol^−1^) surpasses values measured for pristine H_2_BDC (‐26.20 kcal mol^−1^) and amino‐functionalized H_2_BDC (‐26.64 kcal mol^−1^) by over two‐fold, demonstrating the significant enhancement in lithium polysulfide chemisorption conferred by ‐SO_3_H functionalization. Furthermore, the red dashed lines represent Li─O bonds, and the shorter bond lengths indicate stronger interactions. HSO_3_‐H_2_BDC shows two Li─O bonds (2.05 and 2.01 Å), demonstrating a stronger binding force on LiPS. The black dashed lines represent the van der Waals surface mutual penetration distance, where larger values typically indicate stronger interactions. Clearly, HSO_3_‐H_2_BDC exhibits a stronger interaction with Li_2_S_4_. Figure 1c displays the Li^+^@H_2_BDC, Li^+^@NH_2_‐H_2_BDC, and Li^+^@HSO_3_‐H_2_BDC ion‐pairing configurations. Black dashed lines delineate the effects of H_2_BDC, amino‐functionalized H_2_BDC, and sulfonic acid‐functionalized H_2_BDC upon Li^+^ transport. Performance hierarchy reveals sulfonic acid‐functionalized H_2_BDC > amino‐functionalized H_2_BDC > H_2_BDC, signifying superior Li^+^ mobility and distribution uniformity in sulfonic acid‐modified frameworks. The Li^+^@HSO_3_‐H_2_BDC ion‐pairing configuration exhibits the highest binding energy (‐157.80 eV), further confirming its ability to accelerate Li^+^ transport. However, the strong adsorption capability of ‐HSO_3_ may hinder the conversion of LiPS, potentially leading to slower reaction kinetics.^[^ ^35^ ^]^

**Figure 2:**
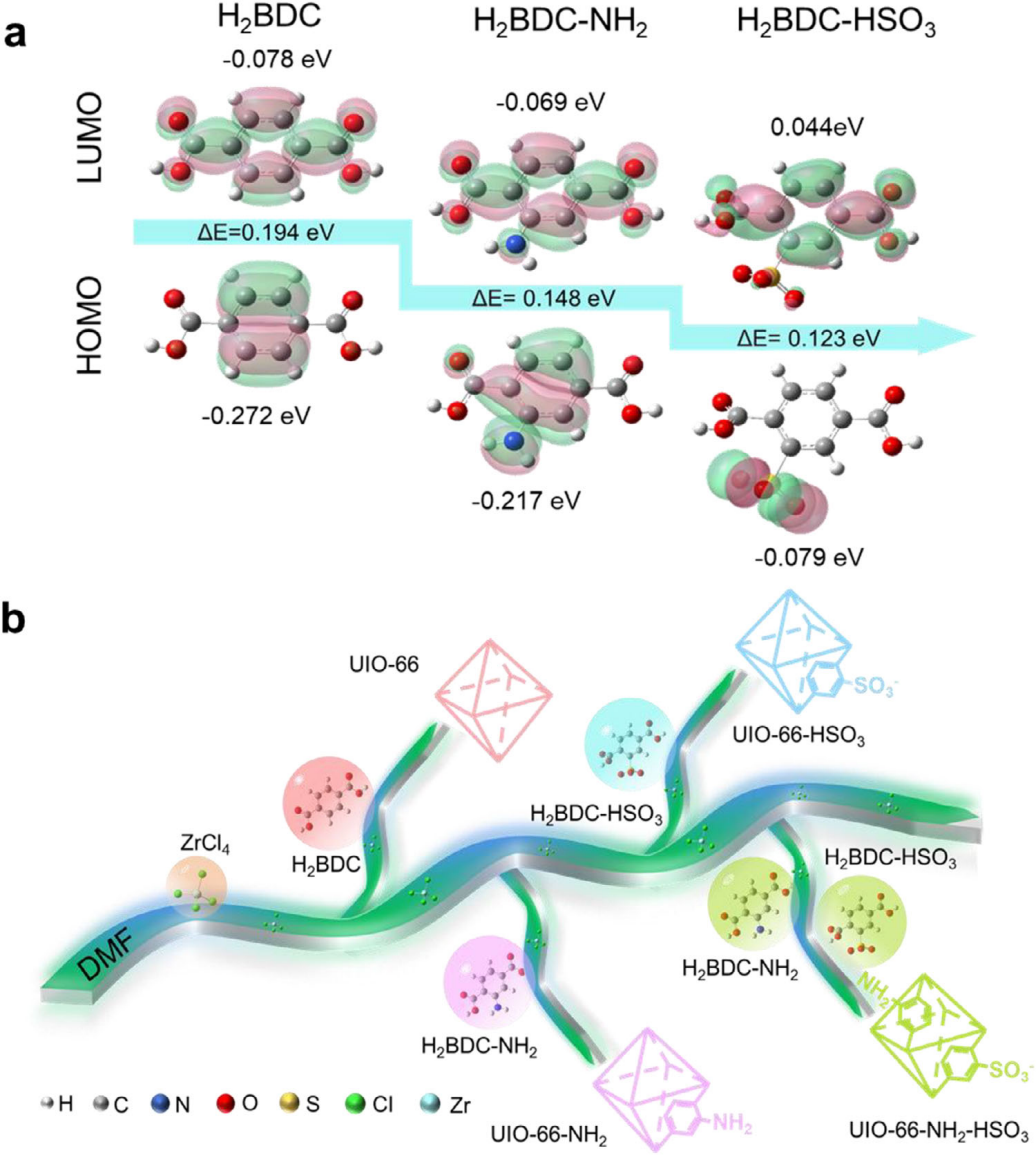
a) The HOMO and LUMO energy levels of H_2_BDC, NH_2_‐H_2_BDC, and HSO_3_‐H_2_BDC. b) Schematic diagram of the preparation process.

**Figure** **2**a shows the highest occupied molecular orbital (HOMO)/lowest unoccupied molecular orbital (LUMO) energies and energy gaps (ΔE) of H_2_BDC, NH_2_‐H_2_BDC, and HSO_3_‐H_2_BDC calculated by DFT methods.^[^ ^36^, ^37^ ^]^ It should be noted that the frontier orbital levels of the ligands determine their electronic conductivity. The HOMO energy order is HSO_3_‐H_2_BDC (‐0.079 eV) > NH_2_‐H_2_BDC (‐0.217 eV) > H_2_BDC (‐0.272 eV), and the LUMO energy order is HSO_3_‐H_2_BDC (0.044 eV) > NH_2_‐H_2_BDC (‐0.069 eV) > H_2_BDC (‐0.078 eV). The high energy of HOMO allows electrons to participate in the reaction more easily, accelerating the sulfur release process. A lower LUMO energy indicates a stronger electron‐accepting ability, which facilitates the oxidation of LiPS by promoting electron transfer from LiPS to the catalytic material during charging. Meanwhile, the reduced energy barrier for electron uptake also improves the kinetics of the reduction reaction during discharge, leading to more efficient and complete conversion between LiPS and Li_2_S. Therefore, lower LUMO energy contributes to smoother redox reactions and enhances the battery's discharge performance. The ΔE of H_2_BDC, NH_2_‐H_2_BDC, and HSO_3_‐H_2_BDC displayed a decreasing trend of 0.194, 0.148, and 0.123 eV, respectively. Suitable HOMO and LUMO energy levels can enhance the interaction with LiPS, thereby inhibiting their dissolution in the electrolyte and reducing the shuttle effect. Among them, HSO_3_‐H_2_BDC possesses the highest HOMO energy level, which contributes to the effective improvement of electron mobility and exhibits stronger reducing properties. In contrast, the LUMO energy levels of NH_2_‐H_2_BDC and H_2_BDC are significantly lower than those of HSO_3_‐H_2_BDC, indicating that they possess stronger electron acceptance. In addition, the ΔE of H_2_BDC is larger than that of NH_2_‐H_2_BDC, indicating a lower charge transfer efficiency of H_2_BDC. These theoretical calculation results provide important guidance for the design of high‐performance MOF host materials. In order to synergistically optimize LiPS adsorption (via the high HOMO energy level of HSO_3_‐H_2_BDC) and charge transfer (via the low LUMO energy level of NH_2_‐H_2_BDC), it was planned to simultaneously integrate NH_2_‐H_2_BDC and HSO_3_‐H_2_BDC into MOFs. For macroscopic‐level comparison of amine/sulfonic acid functional group effects on lithium‐sulfur battery performance, four different MOFs with three ligands (H_2_BDC, NH_2_‐H_2_BDC, and HSO_3_‐H_2_BDC) have been successfully synthesized in Figure 2b and Figure S1 (Supporting Information).^[^ ^38^ ^]^

**Figure 3:**
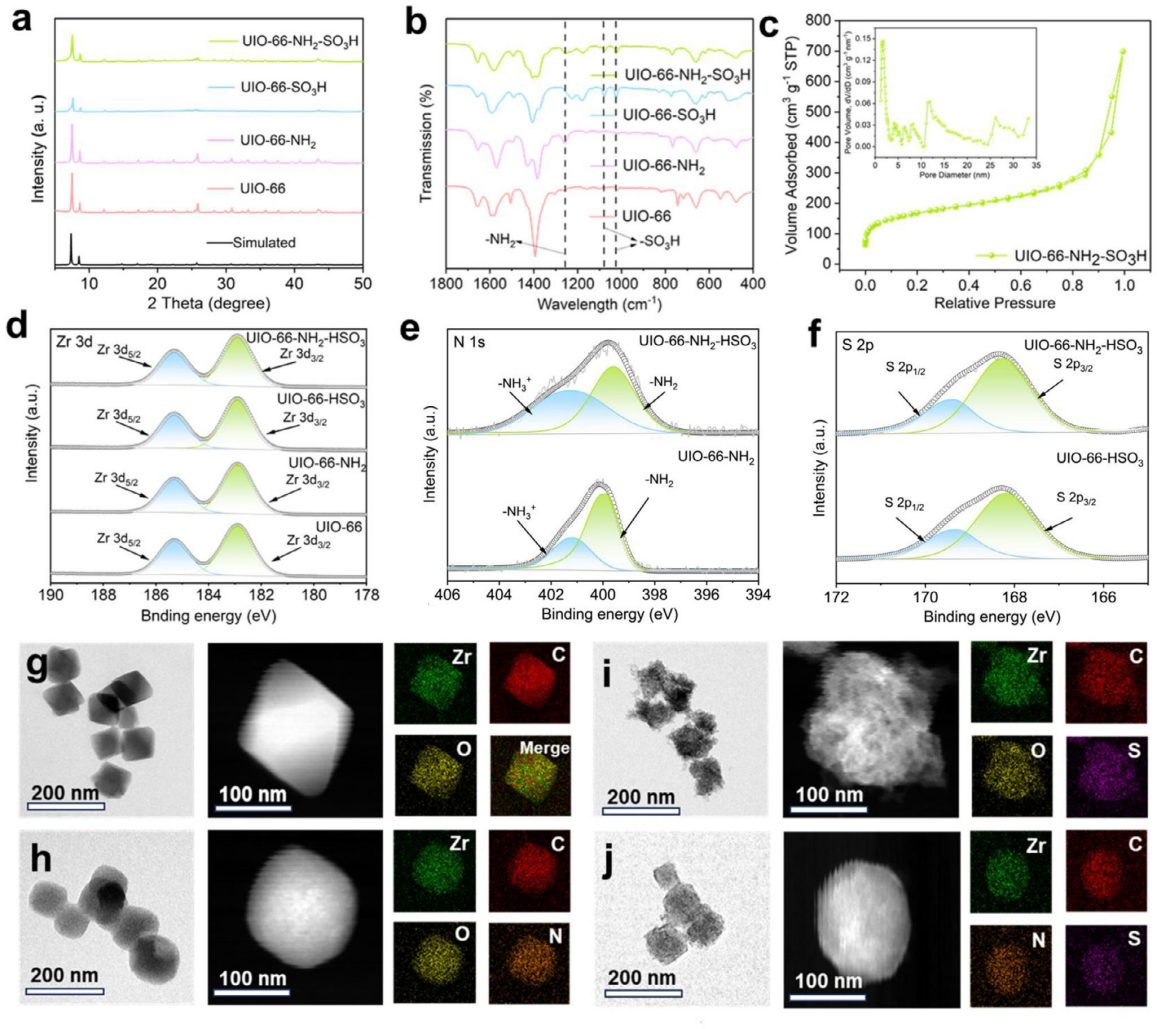
a) XRD patterns and b) FTIR spectroscopy of four samples. c) N_2_ adsorption‐desorption isotherms of dual‐functional MOFs, and the inset is the pore size distribution. High‐resolution XPS spectra of d) Zr 3d, e) N 1s, and f) XPS S 2p spectra of the prepared samples. g–j) HRTEM and elemental mapping images of UIO‐66, UIO‐66‐NH_2_, UIO‐66‐HSO_3,_ and UIO‐66‐NH_2_‐HSO_3_.

X‐ray diffraction (XRD) analysis confirms structural integrity across all synthesized materials, as evidenced by high consistency between experimental patterns and simulated peak positions in **Figures** **3**a and S2 (Supporting Information). The intensities of the UIO‐66‐HSO_3_ and UIO‐66‐NH_2_‐HSO_3_ characteristic peaks are significantly lower than the simulated profiles, which may indicate that the samples are less crystalline with amorphous or partially disordered phases.^[^ ^39^ ^]^ This decrease in crystallinity can be attributed to the introduction of bulky and polar ‐HSO_3_ functional groups, which may disrupt the long‐range periodic order of the MOF, create local defects, or inhibit perfect crystal growth during synthesis. The Fourier Transform infrared spectroscopy (FTIR) analysis in Figure 3b and Figure S2 (Supporting Information) exhibits essentially identical peaks, indicating that MOFs features of UIO‐66 were maintained without significant structural disruption via introducing the ‐SO_3_H and ‐NH_2_. Vibrational modes characteristic of ‐SO_3_H are observed at 1026 and 1079 cm^−1^ in the spectrum. The peak at 1246 cm^−1^ is usually associated with the vibrational mode of ‐NH_2_. Figure 3c and Figure S3 (Supporting Information) present the N_2_ adsorption‐desorption isotherms and corresponding pore size distributions, monofunctional UIO‐66 exhibited large specific surface area (SSA) of 1957.93, 1477.23 m^2^ g.^−1[^ ^40^ ^]^ Although reduced crystallinity often implies lower electronic conductivity, in this case the introduced ‐HSO_3_ groups significantly enhance the ionic conductivity and improve polysulfide adsorption through strong chemical interactions. These functional groups provide abundant binding sites for lithium ions and facilitate rapid Li^+^ transport, thereby compensating for any loss of long‐range order and ultimately enhancing electrochemical performance. The high specific surface area of MOF materials can promote electron conduction and provide more active sites, contributing to the efficient binding of Li^+^ and sulfur. Particularly, following sulfonic acid group functionalization, the modified UIO‐66 derivatives exhibit enhanced pore architectures, which can provide more diffusion channels for Li^+^ and reduce the diffusion resistance of Li^+^ in LSBs.

X‐ray photoelectron spectroscopy (XPS) analysis of four samples was carried out to clarify the elemental composition and chemical bonding.^[^ ^41^, ^42^ ^]^ Figure S4 (Supporting Information) displays the survey spectra of the UiO‐66 series. The characteristic peak signals of N 1s were clearly observed for amino‐functionalized sample, and the characteristic peak signals of S 2p were also observed for sulfonic‐acid‐functionalized sample, indicating the successful introduction of the amino and sulfonic acid groups into UIO‐66. Deconvoluted C 1s spectra of the four MOF samples reveal three characteristic binding energies: 284.8 eV (assignable to C─C bonds), 286.3 eV (attributable to C─O species), and 288.7 eV (corresponding to carbonyl groups), as evidenced in Supporting Information Figure S5 (Supporting Information). The Zr 3d spectrum (Figure 3d) displays characteristic doublet components at binding energies of 182.9 and 185.3 eV, corresponding to the Zr 3d_3/2_ and Zr 3d_5/2_ spin‐orbit states. Meanwhile, XPS analysis of amino‐functionalized sample reveals distinct N 1s signals at 399.8 and 401.2 eV, indicating the presence of ‐NH_2_ and protonated ammonium (‐NH_3_^+^) species, respectively (Figure 3e). Notably, for the bifunctional UIO‐66‐NH_2_‐HSO_3_ material, the N 1s peak exhibits a discernible positive shift (increase in binding energy), which can be attributed to the electron‐withdrawing effect of the adjacent ‐SO_3_H group. This electronegative group reduces the electron density around the nitrogen atom, thereby increasing its core‐level binding energy and confirming a strong electronic interaction between the two functional groups. The S 2p spectra of sulfonic‐acid‐functionalized sample in Figure 3f show two different peaks (S 2p_3/2_ 167.7 eV and S 2p_1/2_ 168.8 eV), which can be attributed to the S─O and S═O bonds. These two bonds indicate that S is bonded to the MOFs mainly in the form of ‐SO_3_H.

The scanning electron microscopy (SEM) and high‐resolution transmission electron microscopy (HRTEM) images of UIO‐66 in Figure 3g and Figure S6a (Supporting Information) show a regular ortho‐octahedral shape with a smooth surface.^[^ ^43^ ^]^ The smooth surface may indicate a high degree of order and perfect crystallinity of the crystals. The particles are usually homogeneous, indicating good crystal growth during synthesis. In Figure 3h and Figure S6b (Supporting Information), UIO‐66‐NH_2_ continues to demonstrate an ortho‐octahedral shape, but the prisms are on the rounded side. The introduction of amino groups may lead to the creation of certain defects or stresses during crystal growth, which makes the prisms less sharp. This may be attributed to the change in the rate of crystal growth due to the presence of ‐NH_2_. In Figure 3i and Figure S6c (Supporting Information), the ortho‐octahedral profile of UIO‐66‐HSO_3_ is shown to be more blurred, indicating that the integrity of the crystals may have been compromised. The introduction of ‐HSO_3_ may have led to irregular growth of the crystals, which produced more amorphous phases or defects during the synthesis, resulting in a decrease in surface smoothness. In Figure 3j and Figure S6d (Supporting Information), it is displayed that UIO‐66‐NH_2_‐HSO_3_ still maintains an ortho‐octahedral shape, but with rounded corners and blurred contours. Co‐functionalization with amino and sulfonic acid groups may induce intricate crystallization pathways featuring elevated defect concentrations and lattice disarray, consequently altering crystalline integrity and surface topology. X‐ray (EDX) elemental mapping showed that N, S, C, O, and Zr were uniformly distributed in samples (Figure 3g–j).^[^ ^44^ ^]^

**Figure 4:**
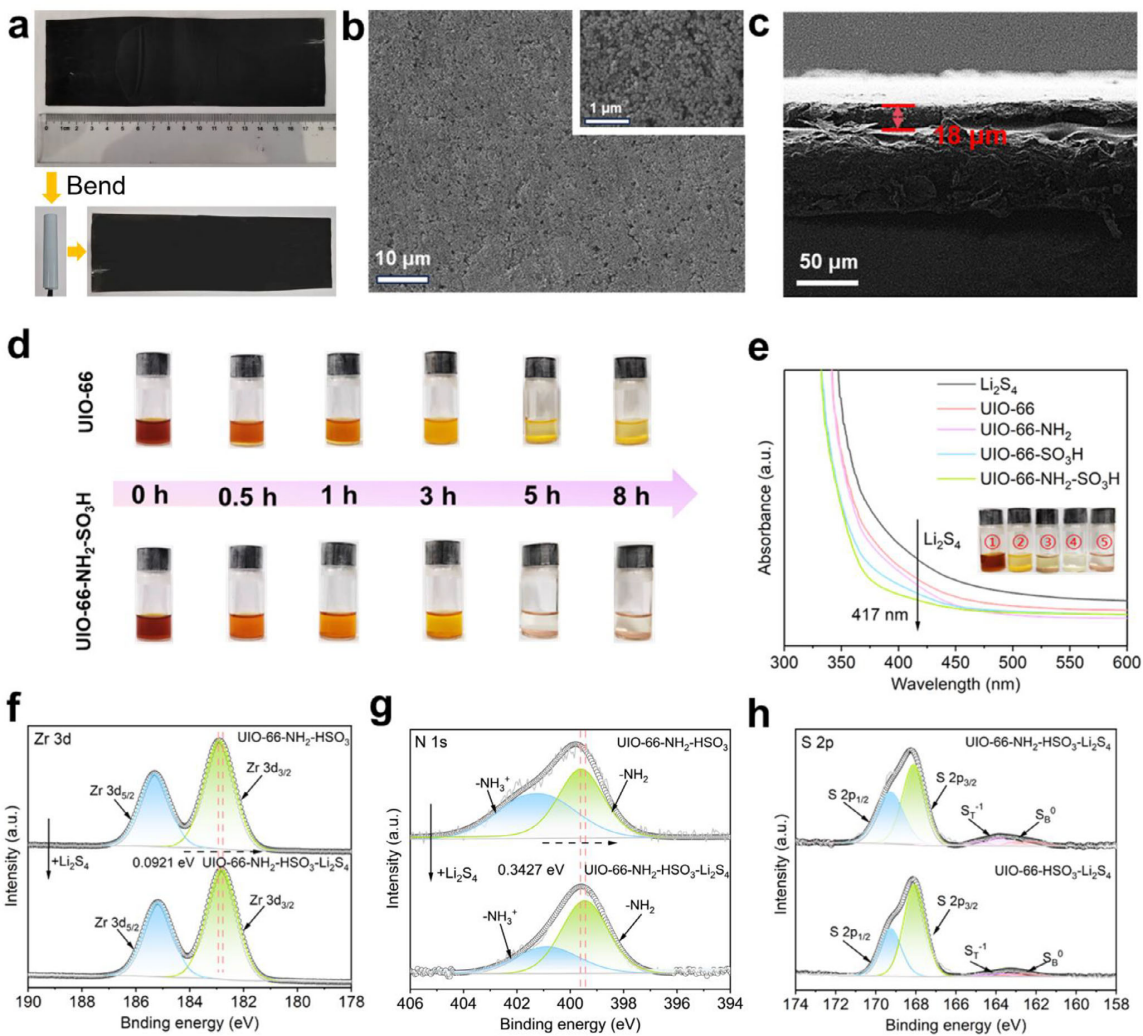
a) Photographs of the separator. SEM images of b) surface and c) Cross‐section of dual functional MOF modified separator membrane. d) Optical photographs of Li_2_S_4_ solution permeation tests at different times. e) UV–vis spectra of Li_2_S_4_ solution before and after adsorption. High‐resolution XPS spectra of f) Zr 3d and g) N 1s with adsorption of Li_2_S_4_. h) XPS spectra of S 2p.

Aforementioned UIO material was coated on the pristine PP separators to form a functionalized surface modification (Figure S7, Supporting Information). The excellent mechanical properties were confirmed by the fact that UIO‐66‐NH_2_‐HSO_3_@PP remained intact after multiple folding, which indicated the ideal bonding of the coating to the pristine separator substrate in **Figure** **4**a.^[^ ^45^ ^]^ The intrinsic porosity of unmodified PP separators, featuring sub‐200 nm pore dimensions (≈100 nm average), enables selective Li^+^ conduction while permitting LiPS species migration across the cathode‐anode interface. SEM images (Figure 4b; Figure S8, Supporting Information) showed that all four samples could completely cover the surface of the macroporous pristine PP separators, which formed a smooth, homogeneous, and dense coating on their surfaces. This facilitates the inhibition of the polysulfide shuttle. Transverse microstructure analysis confirmed an 18 µm functional layer thickness in UIO‐66‐NH_2_‐HSO_3_ composites (Figure 4c), with dimensional consistency validated through instrumental metrology (Figure S9, Supporting Information).

To comparatively assess polysulfide capture performance across functionalized UIO variants, equilibrium adsorption experiments were conducted on dual functional systems (Figure 4d; Figure S10 (Supporting Information).^[^ ^46^ ^]^ The brown Li_2_S_4_ solution turned transparent owing to chemisorption. The results of the adsorption capacity of the UIO samples were pristine UIO‐66 < amine‐functionalized < sulfonic acid‐modified < dual‐functionalized. As an unfunctionalized substrate, UIO‐66 performs the worst in polysulfide adsorption, mainly due to the lack of any functional group that can enhance the adsorption. UIO‐66‐NH_2_ is able to anchor polysulfide anions to some extent with the introduction of ‐NH_2_, but the adsorption capacity is relatively weak due to the lack of negative. Differently, the ‐HSO_3_ can effectively adsorb positively charged polysulfide molecules due to its negative charge and obviously enhance the residence time on the material surface through electrostatic attraction. Therefore, the adsorption ability of UIO‐66‐HSO_3_ on LiPS is stronger than that of UIO‐66‐NH_2_. Consequently, the dual functional UIO‐66 derivative demonstrated optimal polysulfide capture capability, attributable to cooperative interactions between amino and sulfonic moieties. The absorption peak at 417 nm corresponds to the Li_2_S_4_ species, and the efficient adsorption of LiPS is further confirmed by the lowest absorption peak at 417 nm in the UV‐visible absorption spectrum of UIO‐66‐NH_2_‐HSO_3_ (Figure 4e).^[^ ^47^ ^]^

The chemical states of the UIO samples before and after adsorption of LiPS were analyzed by XPS, which allowed an in‐depth understanding of the changes in the properties of MOFs materials in LSBs.^[^ ^48^ ^]^ Upon adsorption of Li_2_S_4_, the binding energy of the Zr 3d_3/2_ fitted peak decreased, implying an interaction between Zr^3+^ and the adsorbed lithium polysulfide molecules (Figure 4f; Figure S11a–c, Supporting Information). This shift suggests electron transfer from lithium polysulfides to Zr^3+^, increasing the electron density around the zirconium ions. UIO‐66‐NH_2_‐HSO_3_ showed the largest decrease in binding energy (0.0921 eV). This superior polysulfide adsorption behavior primarily originates from synergistic mechanisms enabled by concurrent amino and sulfonic functionalities in the dual functional framework. The decrease in the binding energy of UIO‐66‐HSO_3_ was also relatively large (0.0689 eV), indicating that the introduction of the ‐HSO_3_ enhanced the adsorption of LiPS. The decrease in the adsorption energies of UIO‐66 and UIO‐66‐NH_2_ was smaller, suggesting that their adsorption of LiPS was relatively weak. The decrease in the binding energy of the N 1s peak indicated the electron transfer of ‐NH_2_ upon adsorption of LiPS (Figure 4g; Figure S11d, Supporting Information). This electron donation from ‐NH_2_ to lithium polysulfides enhances the adsorption through increased Lewis basicity. The binding energy of the N 1s peak in UIO‐66‐NH_2_‐HSO_3_ is largely shifted toward lower energies (0.3427 eV) due to the effect of ‐HSO_3_ on the ‐NH_2_ electronic environment, in which the ‐NH_2_ group exhibits a stronger adsorption capacity for LiPS. In the S 2p spectra of sulfonic‐acid‐functionalized samples, peaks of terminal sulfur (S_T_^−1^) and bridging sulfur (S_B_^0^) were detected (Figure 4h). The presence of S_T_^−1^ and S_B_^0^ indicates that lithium polysulfides undergo adsorption and transformation in these materials. The existence of these sulfur phases is a sign of the transformation from lithium polysulfide to a more stable sulfur phase.

**Figure 5:**
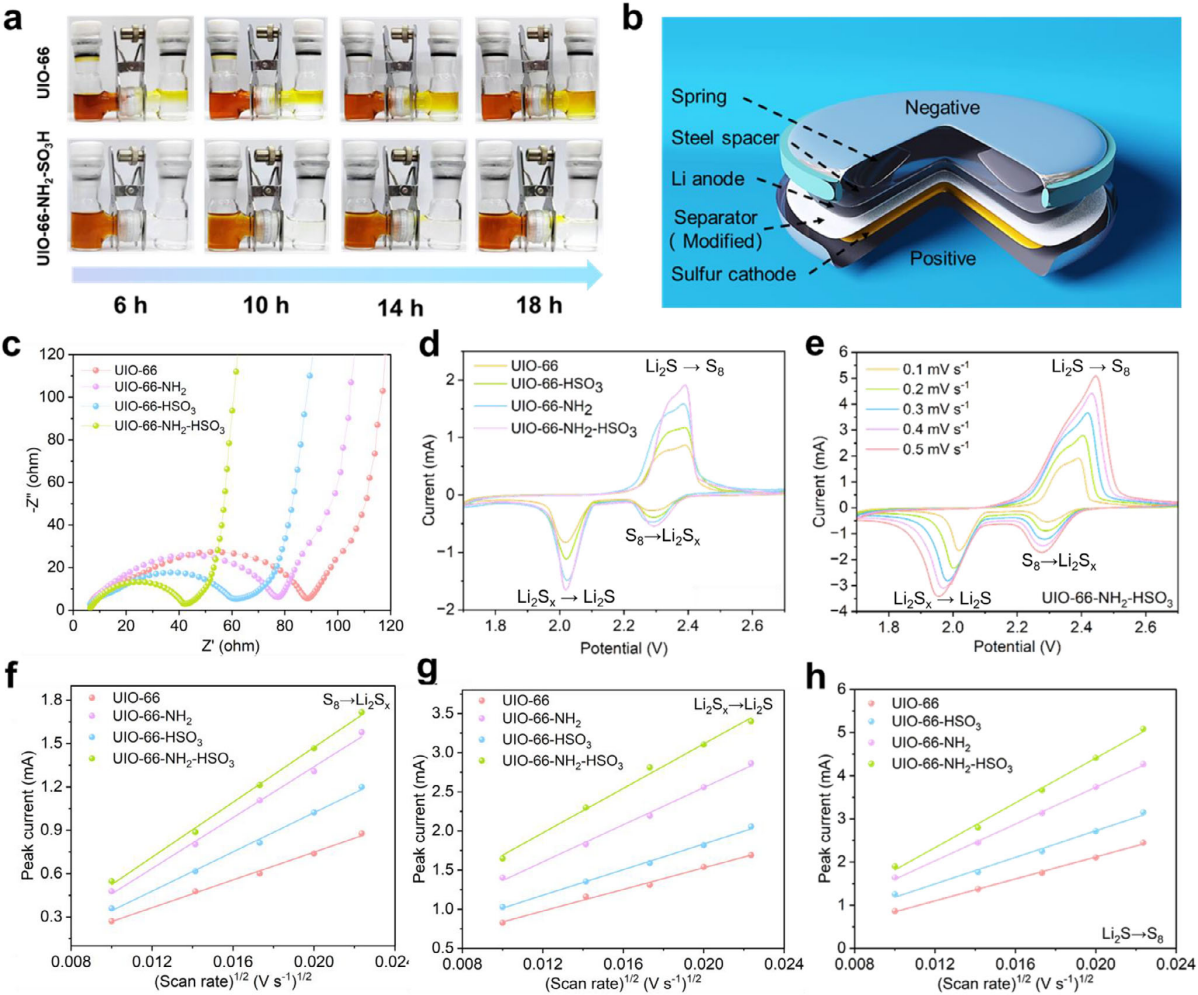
a) LiPS diffusion tests. b) Schematic diagram of coin cells with functionalized separators. c) The EIS spectra and d) first CV cycles of the electrodes. e) CV curves of the electrode at different scan rates. Peak current versus *v* ^0.5^ for f) S_8_ to Li_2_S_x_, g) Li_2_S_x_ to Li_2_S and h) Li_2_S to S_8_.

In an attempt to more visually evaluate the performance of functionalized separators in LSBs in terms of their resistance to LiPS permeation, H‐type electrolytic cell experiments were used to mimic polysulfide diffusion under cycling conditions (**Figure** **5**a; Figure S12, Supporting Information).^[^ ^49^ ^]^ Observing the behavior of various functionalized separators after 18 h of resting, a large amount of LiPS was observed in the right‐side compartment of UIO‐66@PP. It indicated that UIO‐66@PP had poor permeation resistance to LiPS, which resulted in LiPS easily spreading to the other side of the battery during charging and discharging, thus affecting the performance and lifetime of the battery. There was some amount of LiPS permeation in the right compartment of UIO‐66‐NH_2_@PP, but it was reduced compared to UIO‐66@PP. It was suggested that the introduction of ‐NH_2_ enhanced the permeation resistance of the separator to LiPS. The permeation resistance of the UIO‐66‐HSO_3_@PP was further improved with a significant reduction in the amount of LiPS in the right compartment. The ‐HSO_3_ has a negative charge to attract Li^+^ and repel LiPS anions, thus reducing diffusion. As a result, the dual functional MOF modified separator performed almost no significant color changes and almost no penetration of LiPS in the right compartment. Concurrent presence of amino and sulfonic functionalities was shown to substantially amplify interfacial polysulfide blocking efficacy through cooperative mechanisms. Systematic electrochemical characterization of functionalized separator membranes was conducted via CR2032‐coin cell fabrication for LSBs evaluation (Figure 5b). First, the kinetic behavior of four different separators was evaluated using the electrochemical impedance spectroscopy (EIS).^[^ ^50^ ^]^ In the Nyquist plot, the semicircle in the high‐frequency region represents the charge‐transfer resistance (R_ct_), while the diagonal line in the low‐frequency region correlates with the Warburg impedance (Wo). Figure 5c demonstrates that the battery assembled with dual functional MOF modified separator shows the lowest R_ct_, indicating the highest charge transfer efficiency, which is beneficial for the improved multiplication performance and reduced polarization of LSBs. Meanwhile, the lower R_ct_ implies a smoother charge transfer process between the electrode and the electrolyte, which can improve the discharge capability and charge speed of the battery. In the low‐frequency region, the linear characteristics of the Warburg trails are closely related to the diffusion resistance of Li^+^. The Wo slope of the UIO‐66‐NH_2_‐HSO_3_@PP separator is the largest, indicating the rapid Li^+^ diffusion process.^[^ ^51^ ^]^ Cyclic voltammetry (CV) tests were performed on different separators (Figure S13a–d, Supporting Information), and the first three cycle curves exhibit good overlap of the current response curves, meaning an excellent reversibility of these separators. It should be noted that the first CV curve of the battery assembled with dual functional MOF modified separator reveals increased current response and decreased polarization gap (Figure 5d). This pronounced current enhancement corroborates the multifunctional separator's capability to facilitate interfacial charge transfer kinetics while accelerating ion diffusion in redox processes. The smaller polarization voltage indicates its lower internal resistance and excellent reaction kinetics.^[^ ^52^ ^]^ Figure 5e and Figure S14a–c (Supporting Information) demonstrate two distinct reduction peaks that appeared in the CV curves when the batteries were assembled using differently functionalized UIO separators. These reduction peaks are corresponding to the reduction of S_8_ to Li_2_S_x_ and the further reduction of Li_2_S_x_ to Li_2_S, respectively. Moreover, an oxidation peak from Li_2_S to S_8_ was also observed.^[^ ^53^ ^]^ The linear relationship between the peak current and the square root of the scan rate is an important feature in electrochemical studies, and higher values of the slope usually imply larger diffusion coefficients for Li^+^. Batteries incorporating the UIO‐66‐NH_2_‐HSO_3_@PP modified separator exhibit steeper slopes in both their oxidation and reduction peaks (Figure 5f–h). The calculated lithium‐ion diffusion coefficients (D_Li+_) of UIO‐66‐NH_2_‐HSO_3_@PP based on the data in Figure 5f,g are 8.46 × 10^−15^, 1.32 × 10^−14^, and 4.20 × 10^−14^ cm^2^·s^−1^, respectively, which are significantly higher than those of other modified separators. This implies a larger diffusion coefficient of Li^+^ in the material, which indicates favorable Li^+^ conductivity. This contributes to the rapid charging and discharging capabilities of the battery.^[^ ^54^ ^]^

**Figure 6:**
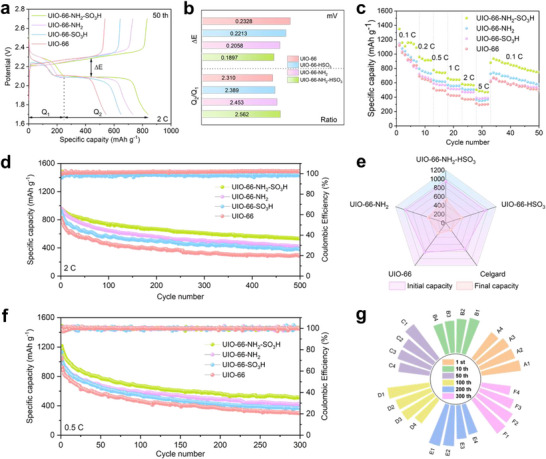
a) Comparison of platform capacity and polarization potential of four electrodes verified by the Galvanostatic charge‐discharge profiles. b) Further comparison of the voltage polarization gap and capacity ratios of the electrodes. c) Specific capability of the electrodes at different current densities. d) Long‐term cycling performance and the corresponding capacity retention analysis via radar chart e). f) Comparison of the cycling performance at 0.5 C. g) ΔE at 0.5C in cycle, the A‐F represent different numbers of cycles, while 1–4 correspond to four different separator materials: UIO‐66@PP, UIO‐66‐NH_2_@PP, UIO‐66‐HSO_3_@PP, and UIO‐66‐NH_2_‐HSO_3_@PP.

In Figure S15a–d (Supporting Information), the electrostatic charge‐discharge (GCD) curves for different cycles at a current density of 2 C clearly indicate two discharge plateaus that correspond to the conversion of S_8_ to Li_2_S_x_ and then to Li_2_S.^[^ ^55^ ^]^ In **Figure** **6**a, the GCD curves of different separators in LSBs at 2 C can be observed. These curves displayed distinct high discharge plateaus (Q_1_) and low discharge plateaus (Q_2_), quantitatively correlating with the multi‐phase evolution during sulfur redox reactions in LSBs. The kinetic properties of the redox reactions of LSBs were quantitatively evaluated by the voltage polarization gap (ΔE) and the capacity ratio (Q_2_/Q_1_). Battery assembled with dual functional MOF modified separator exhibits the highest Q_2_/Q_1_ value (2.562) and the lowest ΔE value (189.7 mV) in Figure 6b. The high Q_2_/Q_1_ ratio signifies enhanced reaction kinetics during the conversion of soluble long‐chain polysulfides to insoluble Li_2_S, which is often the rate‐limiting step due to sluggish nucleation and growth of solid Li_2_S. A larger Q_2_ relative to Q_1_ reflects more complete conversion of intermediates to the final discharge product, suggesting improved sulfur utilization, facilitated Li^+^ diffusion, and more efficient redox kinetics. This indicates that the battery material has favorable electrical conductivity and ion mobility in this process.^[^ ^56^ ^]^ The smaller polarization potential implies lower internal resistance and higher kinetic efficiency, reducing the energy loss of LSBs during charging and discharging. Figure 6c and Figure S16 (Supporting Information) show the specific capacity of the cells assembled with different separators at various current densities. The discharge specific capacities of the cell with UIO‐66‐NH_2_‐HSO_3_@PP were 1350, 962, 778, 663, 585, and 504 mAh g^−1^, respectively, when the current density was gradually increased from 0.1 C to 5 C.^[^ ^57^ ^]^ The performance was significantly better than that of the other separators (Figure S17, Supporting Information), which demonstrated excellent sulfur utilization. When the current density was gradually restored to 0.1 C, UIO‐66‐NH_2_‐HSO_3_@PP was still able to recover to the level of 937 mAh g^−1^. These results demonstrate exceptional tolerance to high current density, good structural stability, and electrochemical reversibility. In Figure 6d, the battery capacity decay rate of the LSBs assembled with UIO‐66‐NH_2_‐HSO_3_@PP is as low as 0.085% at 2 C, indicating that the battery maintains a favorable capacity stability over a long period of time.^[^ ^58^ ^]^ However, compared with dual functional MOF modified separator, the cycling performance of the LSBs assembled with the other three separators are relatively poor, meaning that the dual‐functional MOFs show a superior advantage in the inhibition of intermediated products of LiPS. In addition, Figure 6e and Figure S18 (Supporting Information) demonstrate substantially enhanced cycling stability in functionalized separators versus an unmodified PP separator, with dual functional UIO‐66‐NH_2_‐HSO_3_@PP exhibiting optimal electrochemical performance. Particularly, after 500 cycles charge/discharge process, the battery with UIO‐66‐NH_2_‐HSO_3_@PP membrane still shows an ultra‐high specific capacity of 533.4 mAh g‐1. Moreover, when cycled at 0.5 C, similar results are obtained (Figure 6f; Figures S19 and S20, Supporting Information). First, the batteries assembled with various separator membranes exhibit significantly different initial discharge capacities.^[^ ^59^ ^]^ The initial discharge capacity of the battery with dual functional MOF separator is 1215.7 mAh g^−1^, which is superior to most of the reported results and the assembled batteries with the other three modified separators. In addition, after 300 cycles charge/discharge process, the bar radial plots of ΔE were obtained by calculating the GCD curves (Figure 6g; Figure S21, Supporting Information). The ΔE value of the battery assembled with UIO‐66‐NH_2_‐HSO_3_@PP is always minimized, indicating that it exhibits the best electrochemical performance during the 300 cycles.^[^ ^60^ ^]^ The above results verify that the systematic effect of the dual functional MOF separator is vital for the synergistic resolve of the shuttle effect of LiPS and sluggish reaction kinetics, leading to higher energy conversion efficiency.

**Figure 7:**
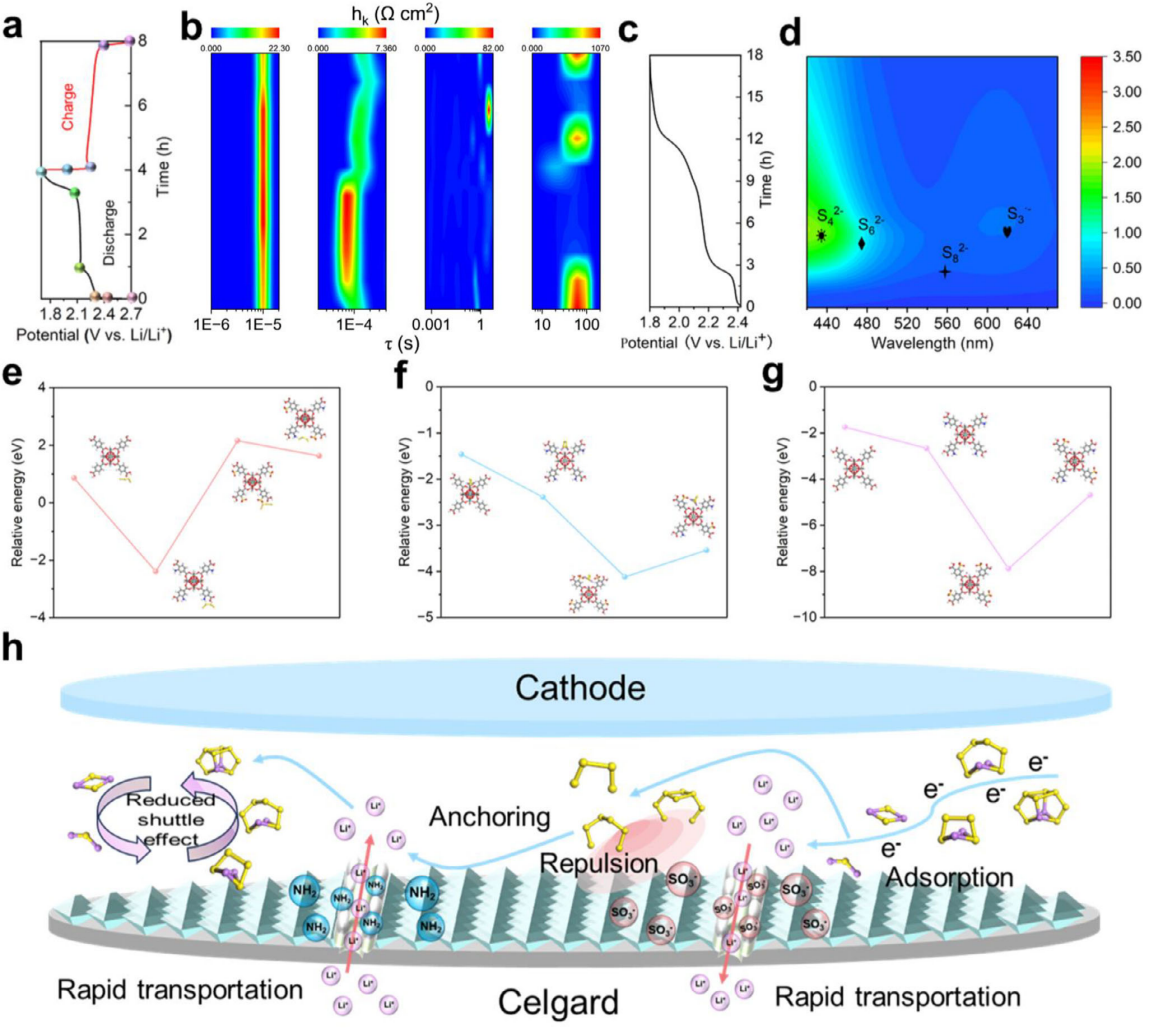
a,b) In situ electrochemical impedance spectroscopy DRT analysis results. c,d) In situ UV–vis spectra. e) The interaction energies of S_4_^2−^. f) Adsorption energy for Li_2_S_4_ molecules. g) The binding energy of Li^+^_._ h) Schematic diagram of the synergistic action of ‐NH_2_ and ‐HSO_3_ in LSBs.

In **Figure** **7**a,b, in situ electrochemical impedance spectroscopy with distribution of relaxation times (DRT) analysis reveals that the dual functional UIO‐66‐NH_2_‐HSO_3_@PP synergistically optimizes charge transfer and ion diffusion kinetics in Li─S batteries. During discharge, the intensified and high‐frequency‐shifted ∼1E^−4^ s peak signifies increased charge transfer resistance (R_ct_) due to polysulfide accumulation and Li^+^ depletion at the cathode interface, while the sharply attenuated ≈100s peak reflects reduced diffusion resistance from liquid‐to‐solid (Li_2_S_4_→Li_2_S) conversion.^[^ ^61^, ^62^ ^]^ During charging, the reversed trends (reduced R_ct_ and dynamically evolving diffusion resistance) demonstrate the separator's dual function, that its sulfonic acid groups suppress polysulfide shuttling to accelerate charge transfer, while enhanced Li^+^ conduction moderates diffusion limitations throughout phase transitions.^[^ ^63^ ^]^ Compared to the unmodified separator, UIO‐66‐NH2‐HSO3@PP exhibits significantly reduced impedance across all major relaxation processes (Figure S23, Supporting Information) In Figure 7c,d, and Figure S22 (Supporting Information), in situ UV–vis spectroscopic analyses of the lithium side were performed to deeply investigate the mechanism of inhibiting LIPS shuttling during the discharge process of dual functional UIO‐66‐NH_2_‐HSO_3_@PP and pristine PP. During the discharge of LSBs, the redox reaction of sulfur is a complex multielectron process involving the generation of multiple intermediates.^[^ ^64^ ^]^ The discharge begins with the reduction of the S_8_ molecule to S_8_^2−^. With the reaction, S_8_^2−^ is further reduced to produce S_6_^2−^ and S_4_^2−^. In addition, S_3_^·−^ is also an important intermediate product, which is generated in the process of further reduction of S_4_.^2‐[^ ^65^ ^]^ The higher the concentration of the intermediate product, the closer the cooler is to red, while the lower the concentration, the closer the cooler is to blue. In the contour plots, it is clearly observed that UIO‐66‐NH_2_‐HSO_3_@PP has an overall blue color, while the pristine PP has an overall reddish color. This occurrence demonstrated that the lithium side of dual functional UIO‐66‐NH_2_‐HSO_3_@PP had lower contents of S_8_^2−^, S_6_^2−^, S_4_^2−^, and S_3_^·−^, which further confirmed the better performance of dual functional UIO‐66‐NH_2_‐HSO_3_@PP in inhibiting the shuttle effect of LIPS. Moreover, the maximum concentration of intermediates in the dual functional separator appeared in a relatively short time, suggesting that it accelerated the reaction kinetics of LIPS conversion.

DFT analysis elucidated how amino and sulfonic functionalizations modulate LIPS and Li^+^ interaction mechanisms throughout LSBs reactions.^[^ ^39^ ^]^ Using S_4_^2−^ as a representative of the LIPS anion, the value of UIO‐66‐NH_2_ is negative, while the value of UIO‐66‐HSO_3_ is positive (Figure 7e; Figure S24, Supporting Information). This indicates that the ‐NH_2_ group has an adsorption effect on S_4_^2‐^ while the ‐HSO_3_ group exhibits a repulsive effect on S_4_^2−^. In Figure 7f and Figure S25 (Supporting Information), using Li_2_S_4_ as a representative of LIPS molecules, four samples all exhibited adsorption on Li_2_S_4_ molecules. Among them, UIO‐66‐HSO_3_ exhibits the strongest adsorption effect at ‐4.12 eV, whereas dual functional UIO‐66‐NH_2_‐HSO_3_ ranks second in adsorption efficacy (‐3.54 eV). Excessive adsorption may result in slowing down the conversion of LIPS. Therefore, UIO‐66‐NH_2_‐HSO_3_ may show superior performance during the reaction of LSBs. UIO‐66 (‐1.74 eV), UIO‐66‐NH_2_ (‐2.66 eV), UIO‐66‐HSO_3_ (‐7.88 eV), and UIO‐66‐NH_2_‐HSO_3_ (‐4.69 eV) were all capable of accelerating Li^+^ transport (Figure 7g; Figure S26, Supporting Information). In this case, the ‐HSO_3_ group contributes to the fast passage of Li^+^, while the ‐NH_2_ group results in a more homogeneous Li^+^ transport. The synergistic effect between these two shows favourable results in LSBs. The proposed entire energy storage mechanism is illustrated in Figure 7h. To begin with, ‐HSO_3_ can strongly adsorb LIPS (Li_2_S_8_, Li_2_S_6_, Li_2_S_4_, Li_2_S_2_, and Li_2_S) in the electrolyte to the surface of the separator at the cathode side. This adsorption allows the Li^+^ in the LIPS to rapidly migrate through the separator from the sulfur positive side to the lithium negative side. On the other hand, polysulfide anions (S_8_^2−^, S_6_^2−^, S_4_^2−^) are repelled by ‐HSO_3_. In addition, ‐NH_2_ anchors the repelled polysulfide anions as well as adsorbing Li^+^ from the lithium anode side to the sulfur anode side. Both of these combine to form stable LIPS. This synergistic effect not only promoted the rapid conversion of LIPS and suppressed the shuttle effect but also effectively formed a rapidly transporting channel for Li^+^, thereby improving the overall performance of LSBs.

## 3 Conclusion

In summary, to investigate the operation mechanism of functional groups anchored in the MOF walls toward the energy storage process of LSBs, four diverse nanomaterials were systematically synthesized. The lithophilic sites were predicted by theoretical calculations, and the corresponding adsorption energy of different groups toward Li^+^ and LiPS was also performed. Although HSO_3_‐H_2_BDC exhibits the maximum adsorption energy for Li^+^ and LIPS, the strong adsorption may adversely affect the desorption and subsequent transfer kinetics. Furthermore, due to the relatively higher HOMO energy of HSO_3_‐H_2_BDC and lower LUMO energy of NH_2_‐H_2_BD displays, the prepared UIO‐66‐NH_2_‐HSO_3_ with dual functionality demonstrates both excellent reaction kinetics and effective inhibition of LiPS shuttle effect. The conclusion was further verified by static adsorption, LIPS shuttle tests, and electrochemical experiments. Meanwhile, the reaction mechanism of UIO‐66‐NH_2_‐HSO_3_ separator during the charge/discharge process of LSBs was investigated by using in situ electrochemical impedance spectroscopy, DRT analysis, in situ UV–vis spectra, and DFT calculations. Through in‐depth analysis for the operation mechanism of dual function groups on Li^+^ and LIPS, we not only reveal the potential of these functional groups in increasing the Li^+^ transport efficiency but also provide guidance for the structural design of MOFs.

## Conflict of Interest

The authors declare no conflict of interest.

## Supporting information



Supporting Information

## Data Availability

The data that support the findings of this study are available from the corresponding author upon reasonable request.
