# Supplementary material for: Synergistic Effect of Dual‐Functional Groups in MOF‐Modified Separators for Efficient Lithium‐Ion Transport and Polysulfide Management of Lithium‐Sulfur Batteries
Source: Adv Sci (Weinh). 2025 Sep 19;12(46):e15034. doi: 10.1002/advs.202515034 (PMC12697768; doi:10.1002/advs.202515034)
Supplement: Supplementary file 1 — Supporting Information [file ADVS-12-e15034-s001.docx]

**Supporting Information**

**Synergistic Effect of Dual-functional Groups in MOF-modified Separators for Efficient Lithium-ion Transport and Polysulfide Management of Lithium-sulfur Batteries**

*Zheng Liu, Wanchang Feng, Haoyang Xu, Zilin Yang, Wenting Li, Mohsen Shakouri, Hsiao-Chien Chen, Fan Zhang*, Huan Pang**

Z. Liu, W. Feng, H. Xu, Z. Yang, W. Li, H. Pang

School of Chemistry and Chemical Engineering, Yangzhou University, Yangzhou, 225002 Jiangsu, P. R. China.

Z. Liu

Key Laboratory of Advanced Energy Materials Chemistry (Ministry of Education), Nankai University, Tianjin 300071, China

F. Zhang

Contemporary Amperex Technology Co., Ltd, Ningde, 352100, China.

M. Shakouri

Canadian Light Source Inc., University of Saskatchewan, Saskatoon, S7N 2V3, Canada.

H. Chen

Center for Reliability Science and Technologies, Chang Gung University, Kidney Research Center, Department of Nephrology, Chang Gung Memorial Hospital, Linkou, Taoyuan 333, Taiwan. China

Corresponding author.

*E-mail addresses*: ZhangF46@catl.com (F. Zhang), panghuan@yzu.edu.cn, huanpangchem@hotmail.com (H. Pang)

Content

[1. Experimental Section 5](#_Toc208863152)

[1.1 Chemicals and Materials 5](#_Toc208863153)

[1.2 Synthesis of UIO-66 5](#_Toc208863154)

[1.3 Synthesis of UIO-66-NH_2_ 5](#_Toc208863155)

[1.4 Synthesis of UIO-66-SO_3_H 5](#_Toc208863156)

[1.5 Synthesis of UIO-66-NH_2_-SO_3_H 5](#_Toc208863157)

[1.6 Preparation of modified separators 6](#_Toc208863158)

[1.7 Preparation of cathode 6](#_Toc208863159)

[1.8. Characterization 6](#_Toc208863160)

[1.9 Adsorption and soaking tests 7](#_Toc208863161)

[1.10 Visualized permeation test 7](#_Toc208863162)

[1.11 Calculation Details 7](#_Toc208863163)

[1.12 Electrochemical Measurements 8](#_Toc208863164)

[1.13 In situ EIS measurements 8](#_Toc208863165)

[1.14 In situ UV-vis measurement 9](#_Toc208863166)

[2. Schematic of the preparation of UIO-66-NH_2_- HSO_3_ 10](#_Toc208863167)

[3. FTIR spectroscopy 11](#_Toc208863168)

[4. N_2_ adsorption–desorption isotherms and pore size distribution 12](#_Toc208863169)

[5. XPS Full-scan spectra 13](#_Toc208863170)

[6. High-resolution XPS spectra of C 1s 14](#_Toc208863171)

[7. SEM images 15](#_Toc208863172)

[8. Schematic diagram of mass preparation of modified pp separators 16](#_Toc208863173)

[9. SEM image of separators surface 17](#_Toc208863174)

[10. Photograph of the UIO-66-NH_2_-HSO_3_ modified PP separator 18](#_Toc208863175)

[11. Optical photographs of Li_2_S_4_ solution permeation tests 19](#_Toc208863176)

[12. High-resolution XPS spectra of Zr 20](#_Toc208863177)

[13. LiPS diffusion tests in the H-shaped cells 21](#_Toc208863178)

[14. CV curves of the initial third cycles 22](#_Toc208863179)

[15. CV curves at various scan rates 23](#_Toc208863180)

[16. GCD profiles 24](#_Toc208863181)

[17. Rate performance of the pristine PP 25](#_Toc208863182)

[18. The charge/discharge profiles 26](#_Toc208863183)

[19. Long-term cycle 27](#_Toc208863184)

[20. Radar map of the initial and final capacities of all separators 28](#_Toc208863185)

[21. Long-term cycle performance at 0.5 C 29](#_Toc208863186)

[22. GCD profiles 30](#_Toc208863187)

[23. Contour maps of in situ UV-vis spectra 31](#_Toc208863188)

[24. In-situ electrochemical impedance spectroscopy DRT analysis results of unmodified separator 32](#_Toc208863189)

[24. The optimized configurations for interaction energies of S_4_^2−^ 33](#_Toc208863190)

[25. The optimized configurations for adsorption energy of Li_2_S_4_ molecules 34](#_Toc208863191)

[26. The optimized configurations for binding energy of Li^+^ 35](#_Toc208863192)

# 1. Experimental Section

## 1.1 Chemicals and Materials

All of the reagents used in this experiment were analytical grade and they used without further purifications. Zirconium (IV) chloride (ZrCl_4_, 99.99 %), terephthalic acid (H_2_BDC, 98 %), 2-amino terephthalic acid (H_2_BDC-NH_2_, 99 %) and benzoic acid (C_6_H_5_COOH, 98 %) were purchased from Aladdin Chemical Reagents Co., ltd. (Shanghai, China). Monosodium 2-sulfoterephthalate (H_2_BDC-SO_3_Na, > 98.0 %) was obtained from Shanghai Macklin Biochemical Co., Ltd. (Shanghai, China). N, N-dimethylformamide (DMF, 99.9%), acetic acid glacial (HAC, 99.5 %) and methanol (MeOH, 99.85 %) were purchased from Sinopharm Chemical Reagent Co., ltd. (Peking, China). Deionized water was obtained through a Mili-Q water-purification system.

## **1.2 Synthesis of UIO-66**

In a typical procedure,1.2 mmol ZrCl_4_, 1.2 mmol H_2_BDC, and 32 mmol C_6_H_5_COOH were added to 60 mL DMF. The mixture was processed with stirring for 30 minutes and then transferred to a 100 ml stainless steel autoclave. The autoclave is sealed and heated at 120 °C for 24 hours. Then, the autoclave was cooled to room temperature. The residue of the reaction mixture was collected after centrifugation, washed three times each with DMF and methanol, and dried under vacuum at 80 °C for 12 h to obtain UiO-66.

1.3 Synthesis of UIO-66-NH_2_
2.4 mmol ZrCl_4_ and 2.4 mmol H_2_BDC-NH_2_ were added to a mixture of 17 ml HAC, 3 ml deionized water and 40 ml DMF. Other conditions are the same as the above operation, and finally obtain UIO-66-NH_2_.

1.4 Synthesis of UIO-66-SO_3_H
2.4 mmol ZrCl_4_ and 2.4 mmol H_2_BDC-SO_3_Na were added to a mixture of 17 ml HAC, 3 ml deionized water and 40 ml DMF. Other conditions are the same as the above operation, and finally obtain UIO-66- SO_3_H.

1.5 Synthesis of UIO-66-NH_2_-SO_3_H
2.4 mmol ZrCl_4_, 1.2 mmol H_2_BDC-NH_2_ and 1.2 mmol H_2_BDC-SO_3_Na were added to a mixture of 17 ml HAC, 3 ml deionized water and 40 ml DMF. Other conditions are the same as the above operation, and finally obtain UIO-66-NH_2_ SO_3_H.

## 1.6 Preparation of modified separators

80 mg of UIO-66-NH_2_-SO_3_H powder and 10 mg of Ketjen Black (KB-600)‌ were weighed and ground until homogeneous, then 200 mg of pre-formulated N-methyl-2-pyrrolidone (NMP) solution containing 5 wt.% polyvinylidene fluoride (PVDF) was added and applied to the surface of the PP separator using a tetrahedral spatula (set to a thickness of 100 μm). The separators were dried in a vacuum oven at 80 °C for 12 h. The large separator with MOFs modification was prepared. The separators were cut into 16 mm pieces. The MOF layer loading per separator is approximately 0.5 mg. For comparison, UIO-66@PP, UIO-66-NH_2_@PP and UIO-66-SO_3_H@PP separators were also prepared by the same method.

## 1.7 Preparation of cathode

KB-600/S composite was first prepared to used as the activated material of LSBs through melt-diffusion method (KB-600 and sublimation of sulfur mixed uniformly into the hydrothermal reaction kettle sealed, warmed to 150 ℃ insulation 12h). For the preparation of cathode, KB-600/S, Super P, and NMP solution containing 5 wt.% PVDF (7:2:1 by weight) were milled together to form uniform slurry. Then, the slurry was coated on the carbon coated Al foil and dried in vacuum at 60 ℃ for 12 h. The obtained working electrodes were cut to circular electrode with a diameter of 12 mm. The mass loading of active sulfur was about 1.1-1.3 mg cm^−2^.

## 1.8. Characterization

The XRD patterns were performed by Rigaku MiniFlex 600 with Cu Kα radiation of 40 KV (λ =1.5418 Å). SEM images were obtained by Zeiss-Supra 55 microscopes at an acceleration voltage of 5 KV. HRTEM and EDX elemental mapping scans were recorded using Tecnai G2 F30 S-TWIN at an acceleration voltage of 300 KV. The N_2_ adsorption-desorption isothermals were obtained by Autosorb-IQ3. XPS analysis was carried out using Thermo Scientific ESCALAB 250Xi Xray photoelectron spectrometer with Al Kα radiation as the excitation source. The accurate sulfur mass on each electrode was calculated according to the elemental analysis data from Elementar, VarioELcube Co. (C, H, N, S mode). Fourier transform infrared (FTIR) transmission spectra were obtained on a BRUKER-EQUINOX-55 IR spectrophotometer.

## 1.9 Adsorption and soaking tests

For the adsorption test, 20 mg UIO-66, UIO-66-NH_2_, UIO-66-SO_3_H and UIO-66-NH_2_-SO_3_H were soaked in 3 mL Li_2_S_4_ solution (10 mmol L^-1^). The Li_2_S_4_ solution were prepared in a solvent mixture of 1,3-dioxolane (DOL) and 1,2-dimethoxyethane (DME) (1:1 in volume) according to the reaction equation:

3S + Li_2_S ⇄ Li_2_S_4_

UV-vis spectra of the above solutions (diluted 5 times before testing) were recorded by using a UV2550 instrument (Shimadzu, Japan). The concentration variation of polysulfides in these solutions was detected from the UV-vis spectra.

## 1.10 Visualized permeation test

An H-type transparent cell was used in an argon-filled glovebox, and a Li_2_S_4_ solution was selected as the representative LiPS. The right chamber was filled with pure DOL/DME mixed solvent (1:1 by volume), and the left chamber was filled with the Li_2_S_6_ solution. Both chambers were separated using a prepared separator (UIO-66@PP, UIO-66-NH_2_@PP, UIO-66-SO_3_H@PP and UIO-66-NH_2_-SO_3_H@PP separator, a diameter of separator = 18 mm).

## 1.11 Calculation Details

All the DFT calculations were conducted using the Gaussian 09 software package. The exchange-correlation effects were described by the B3LYP functional. The core-valence interactions were accounted for by the 6-31G(d) basis set for all atoms. The geometric configurations of all molecules and adsorption complexes were fully optimized. The convergence criteria for the optimization were set to the default thresholds of the program (maximum force: 0.000450 Hartree/Bohr, RMS force: 0.000300 Hartree/Bohr, maximum displacement: 0.001800 Å, RMS displacement: 0.001200 Å). Frequency calculations were performed at the same level of theory on the optimized structures to confirm them as true local minima (no imaginary frequencies) or transition states (one imaginary frequency). The empirical dispersion correction (Grimme's GD3) was incorporated to describe the dispersion interactions.

The adsorption energy (Eads) of adsorbate A was defined as

E_ads_ = E_[surface + adsorbate]_ – E_surface_ – E_adsorbate_

in which E_[surface + adsorbate]_, E_surface_ and E_adsorbate_ are the total energies of the substrate with the molecule adsorbed, the substrate and the isolated molecule, respectively.

## 1.12 Electrochemical Measurements

The CR 2032-type coin cells were fabricated using the working electrode, lithium foil as the counter and anode electrode, Celgard 2400 as the separator. The electrolyte was used 1.0 mol L^-1^ lithium bis(trifluoromethanesulfonyl)imide (LiTFSI Sigma-Aldrich (USA), 99.95%) in 1, 3-dioxolane (DOL, Sigma-Aldrich (USA), 99.0%) and 1,2-dimethoxyethane (DME, Sigma-Aldrich (USA), 99.0% (volume ratio, 1:1) with 1 wt.% LiNO_3_ in an argon-filled glove box (where both water and oxygen levels are below 0.1 ppm. The value of the electrolyte to S (E/S) ratio is 15 μL mg^-1^ (according to the weight of S). The rate capability was also tested by varying the current density from 0.1 C to 5 C (1 C = 1675 mAh g^-1^) on a battery measurement system (CT2001A, Wuhan Land, China) at room temperature. CV and EIS curves were measured on an electrochemical workstation (CHI660E, Chenhua, Shanghai, China). CV curves was performed from 2.8 V to 1.7 V (vs Li^+^/Li) at a scanning rate of 0.1 mV s^-1^, and the frequency of EIS was performed form 100 kHz to 0.01 Hz at open-circuit potential.

## 1.13 In situ EIS measurements

The DH7000D electrochemical workstation was used to synchronize the acquisition of in-situ impedance data during constant-current charging and discharging of LSBs. The frequency range of the test was 100 kHz to 0.01 Hz, the amplitude of the AC disturbance was 5 mV (relative to the open-circuit potential), and the voltage window was controlled in the range of 1.7-2.7 V. The impedance was measured at 10 characteristic points during the charging and discharging cycles, keeping the dynamic condition of the battery throughout the test. Ten characteristic points were selected for impedance measurements during the charge/discharge cycle, and the dynamic battery condition was maintained throughout the test to capture the electrode/electrolyte interface evolution behavior. The experiments were conducted at a constant temperature of 25.0 ± 0.5°C, and the battery was allowed to stand for 4 h before testing to ensure that the open-circuit potential was stabilized. The analysis was implemented using a dedicated DRT analysis package in a Python environment. By examining the evolution of the position, intensity, and number of characteristic peaks in the DRT spectra at different states of charge and discharge, we were able to quantitatively track the kinetics of critical processes in Li-S batteries, such as polysulfide shuttling, the formation and evolution of interfacial films (SEI/CEI), charge transfer, and solid-state diffusion.

## 1.14 In situ UV-vis measurement

The cathode slurry comprised of an active material powder KB-600/S, Super P, and PVDF with a mass ratio of 7:2:1. Then, the nickel foam was selected as the collector (1 × 0.6 cm^2^). The sulfur mass loading on the electrode was ~6 mg cm^-2^, and the current density is 0.05 C. In situ UV-vis cells were assembled using KB-600/S electrode as the cathodes, UIO-66-NH_2_-SO_3_H@PP as the separator and Li metal as the anode, using a custom made in situ cuvette. In situ cuvette cell was assembled in an Ar filled glove box and sealed in 3 mL of Li-S electrolyte. UV-vis absorption spectra (UV-vis, Shimadzu UVmini-1280 spectrophotometer) were used to detect the concentration and elemental chemical states of the LiPS.

# 2. Schematic of the preparation of UIO-66-NH_2_- HSO_3_

**
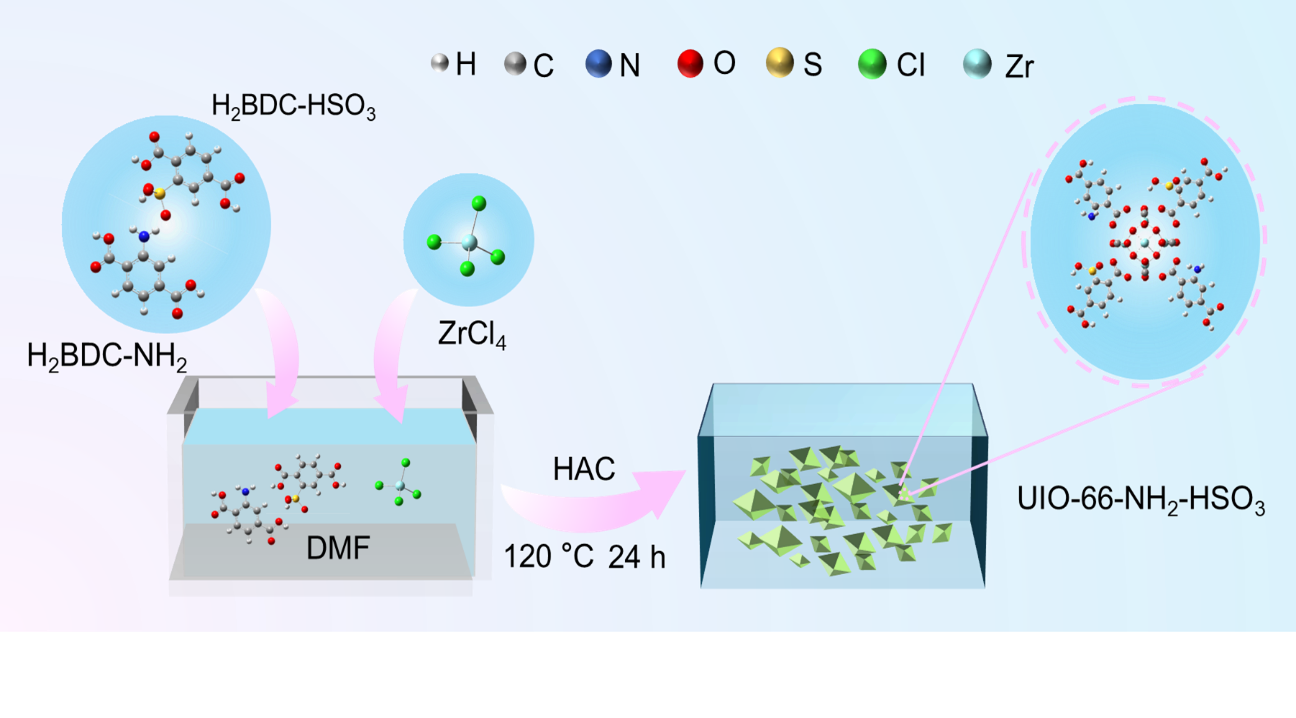
**

**Figure S1.** Schematic of the preparation of UIO-66-NH_2_- HSO_3_.

# 3. FTIR spectroscopy


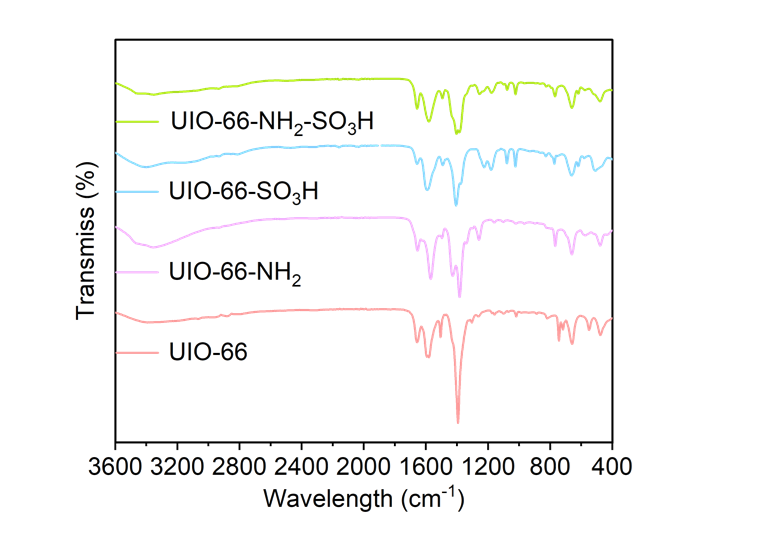
**Figure S2.** FTIR spectroscopy (full spectrum).

# 4. N_2_ adsorption–desorption isotherms and pore size distribution

**Figure S3.** N_2_ adsorption–desorption isotherms and pore size distribution of UIO-66, UIO-66-NH_2_ and UIO-66-HSO_3_.


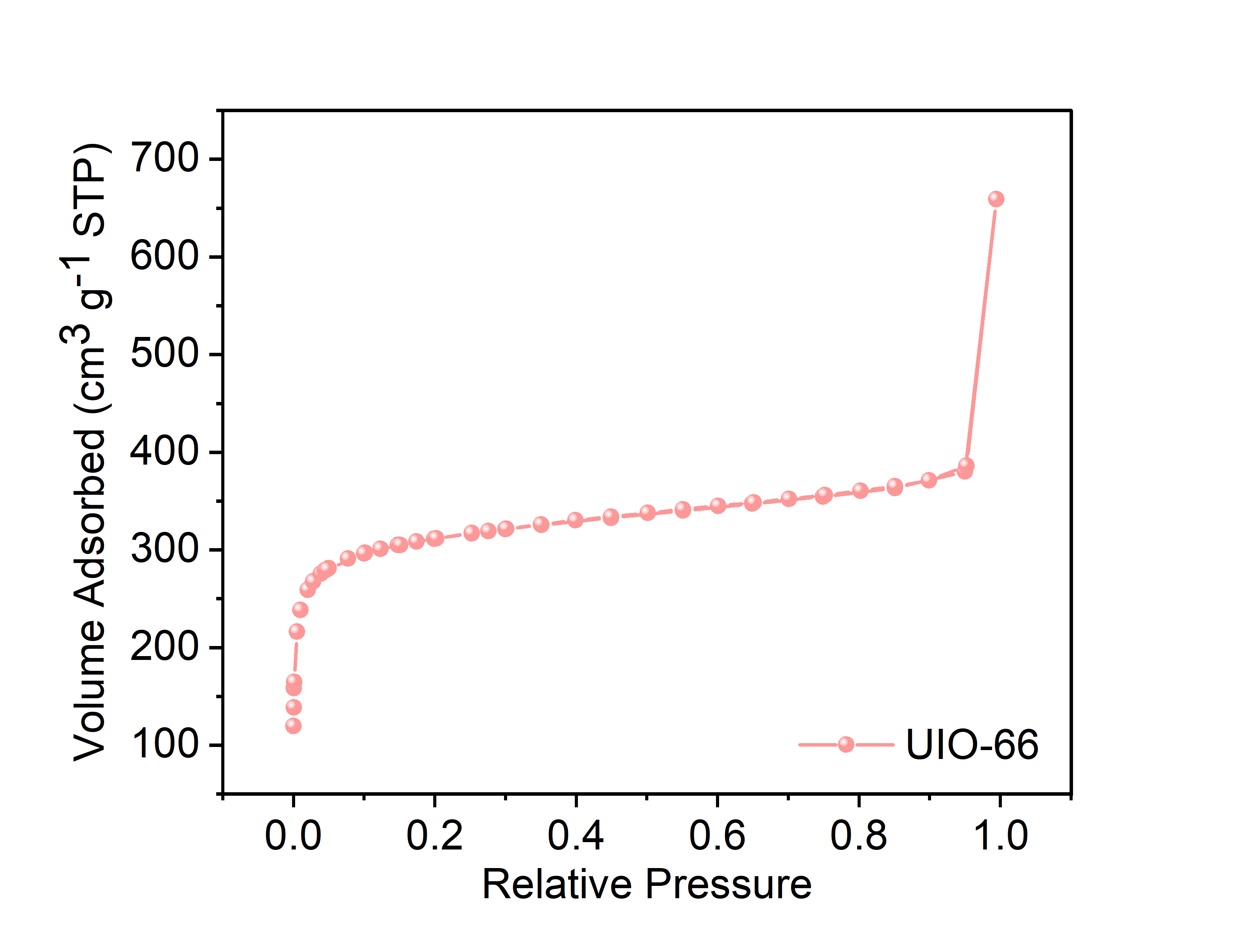

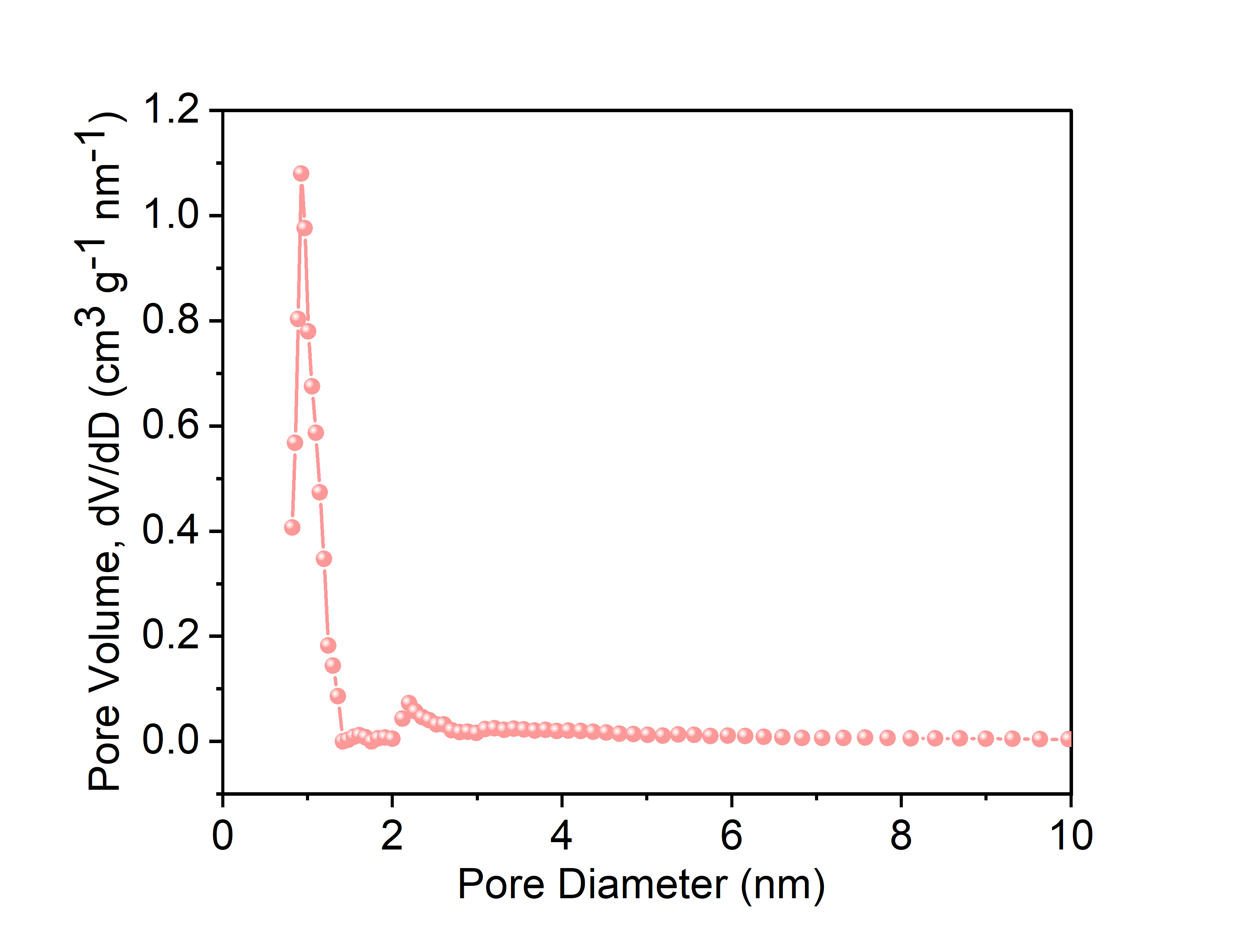

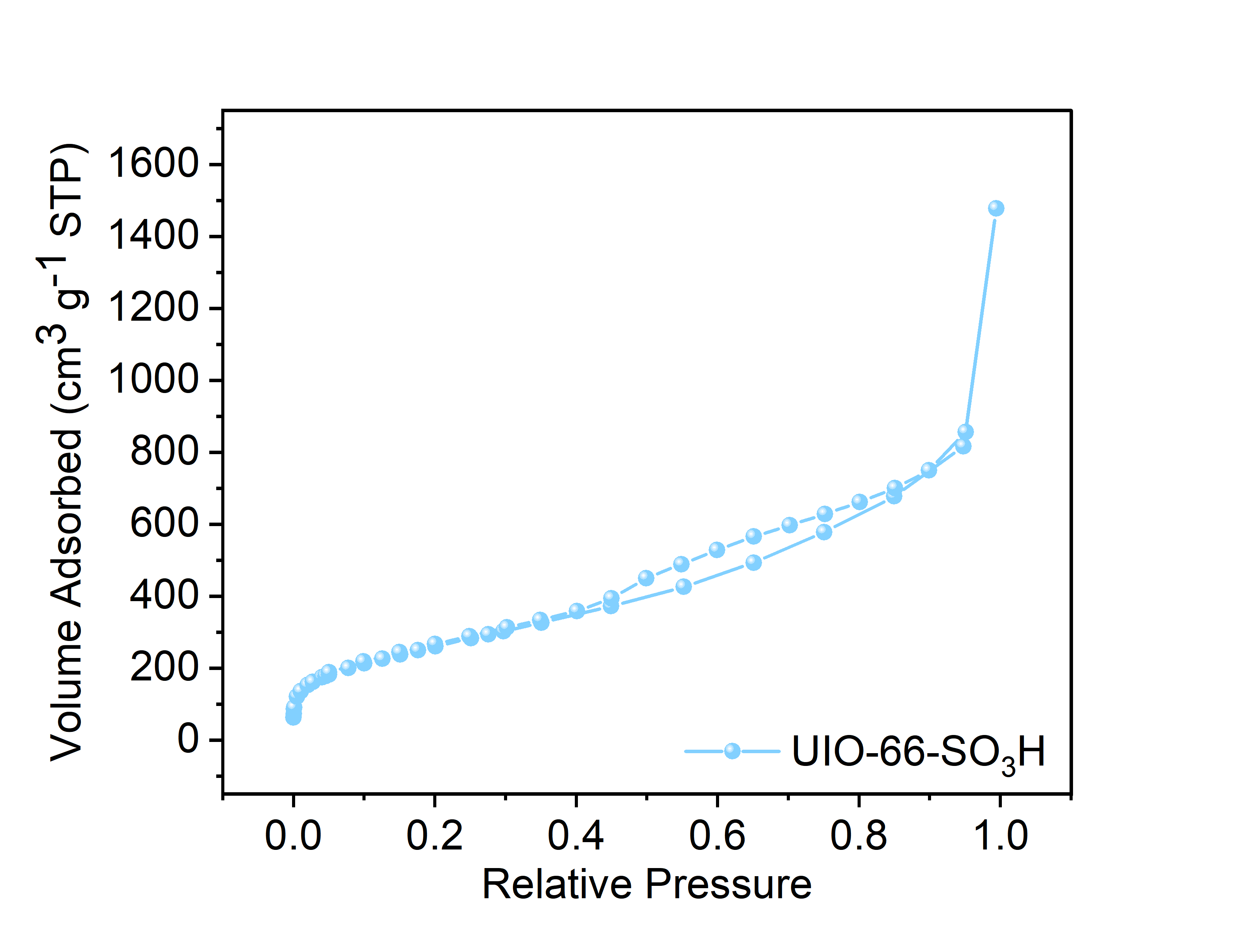

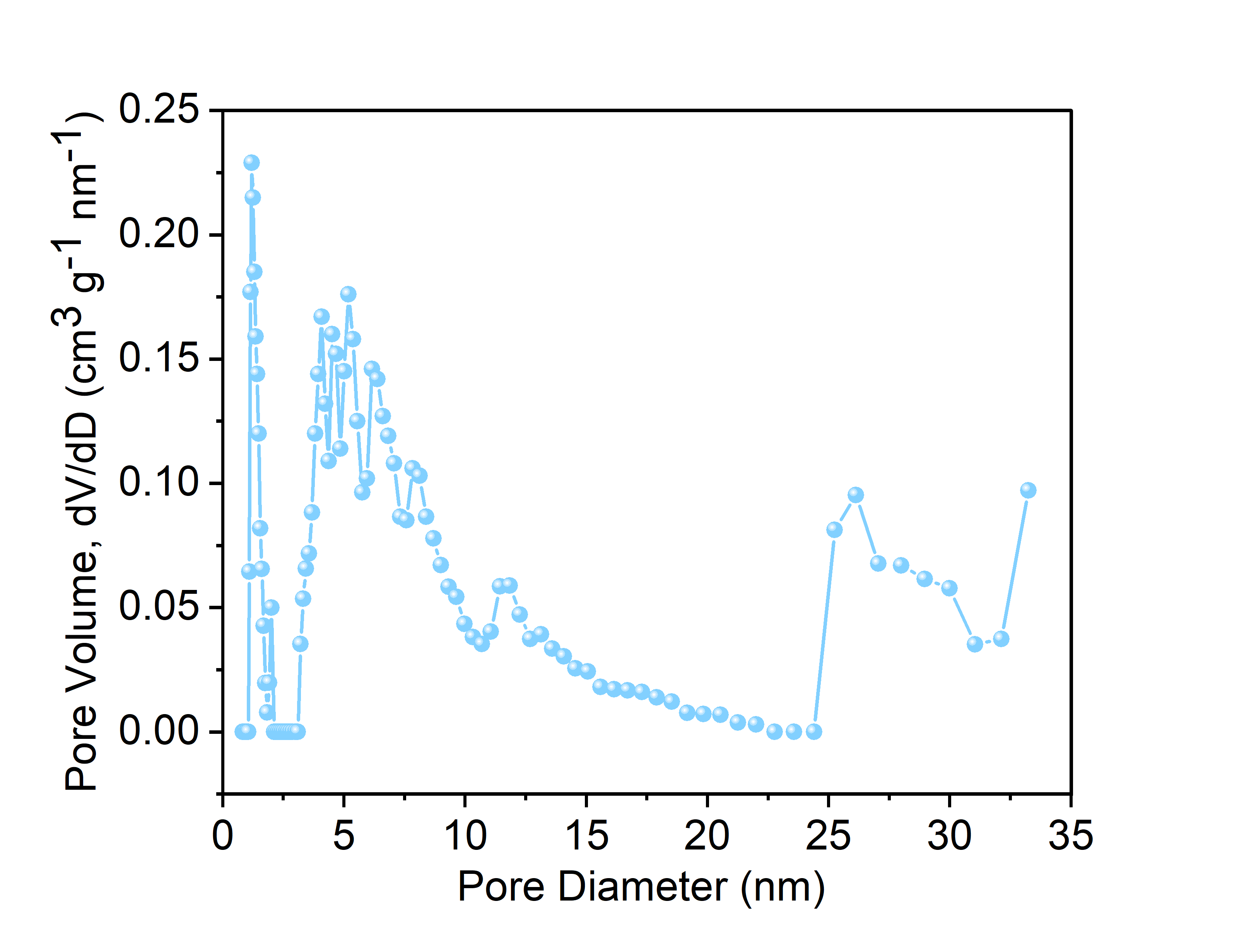

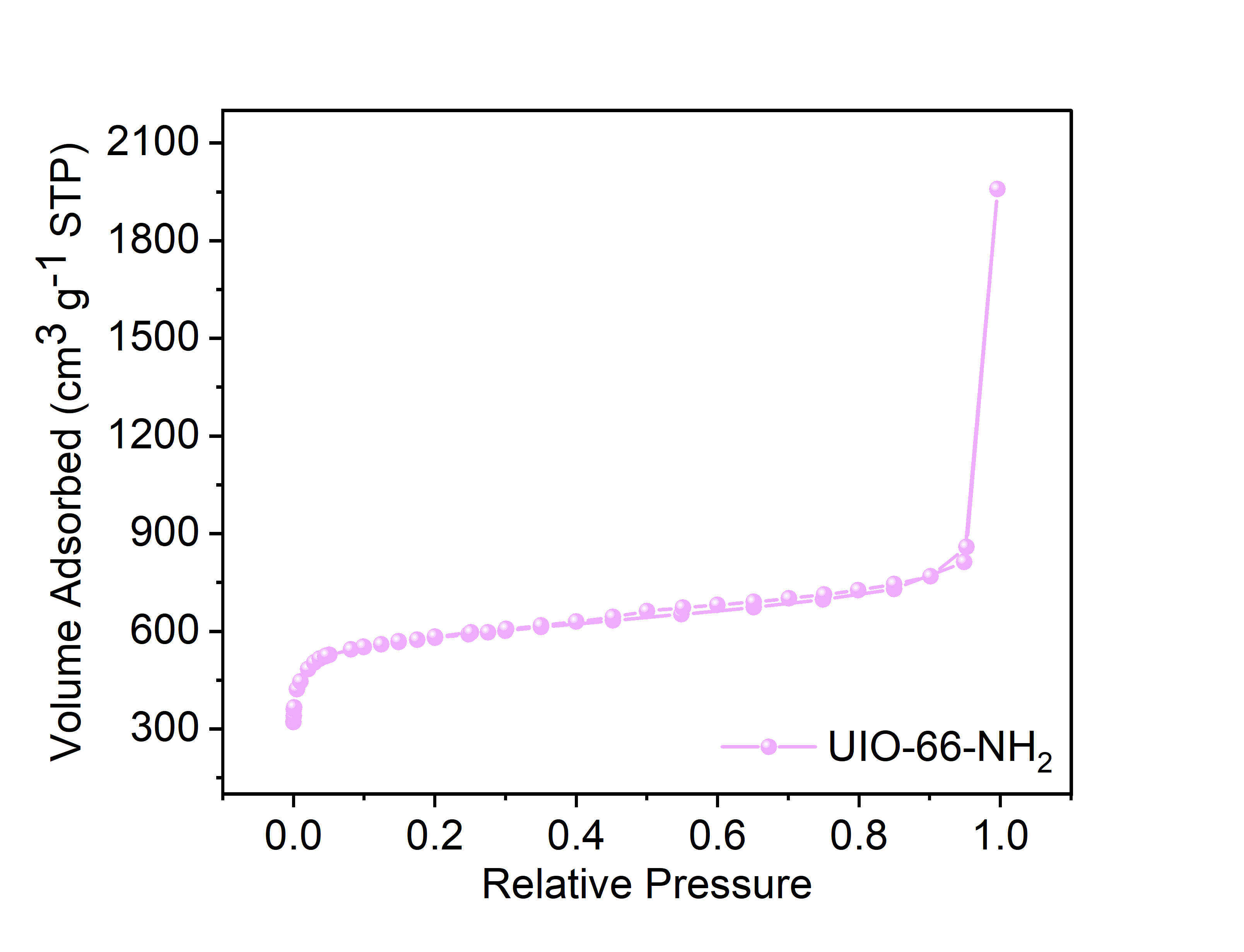

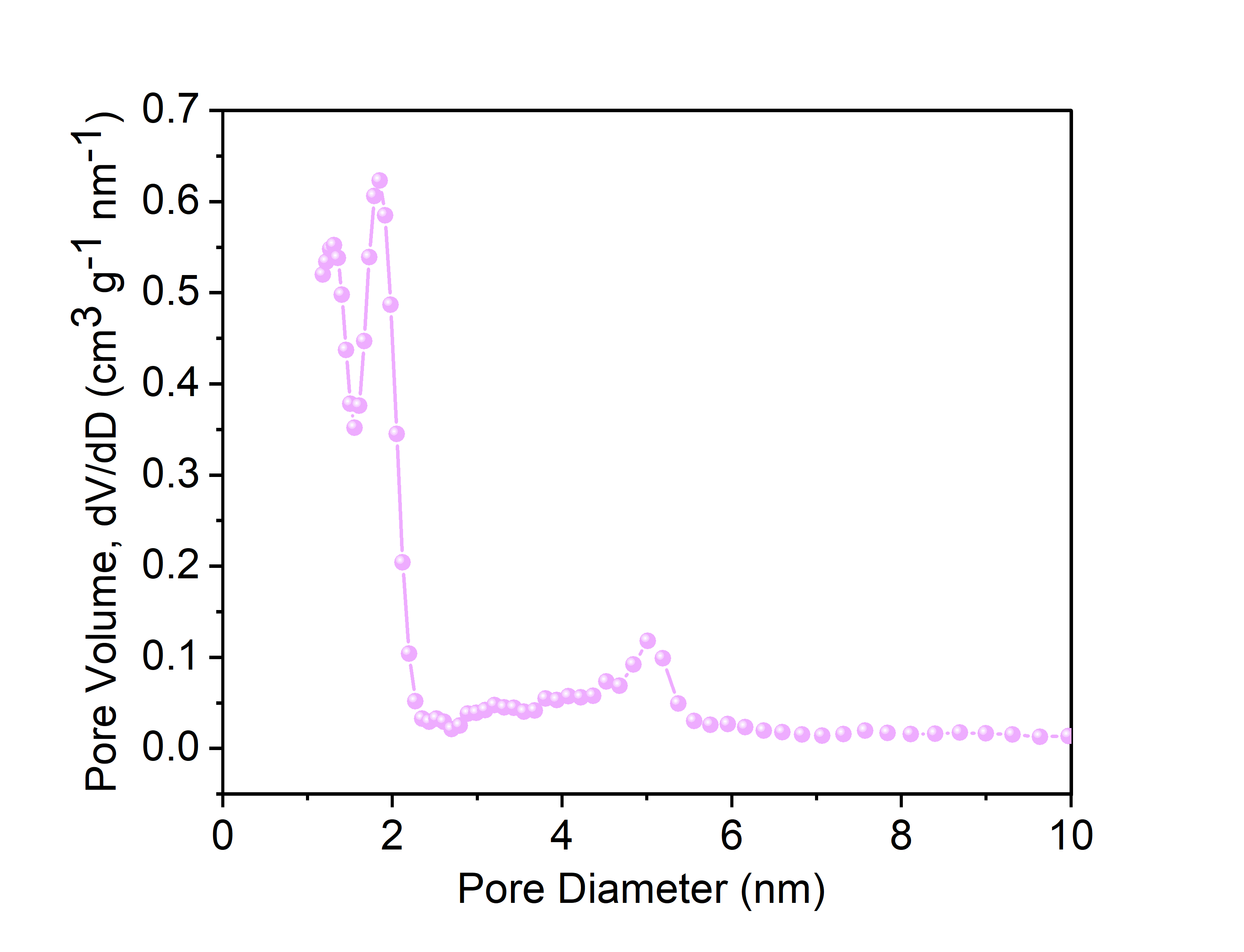


# 5. XPS Full-scan spectra

**
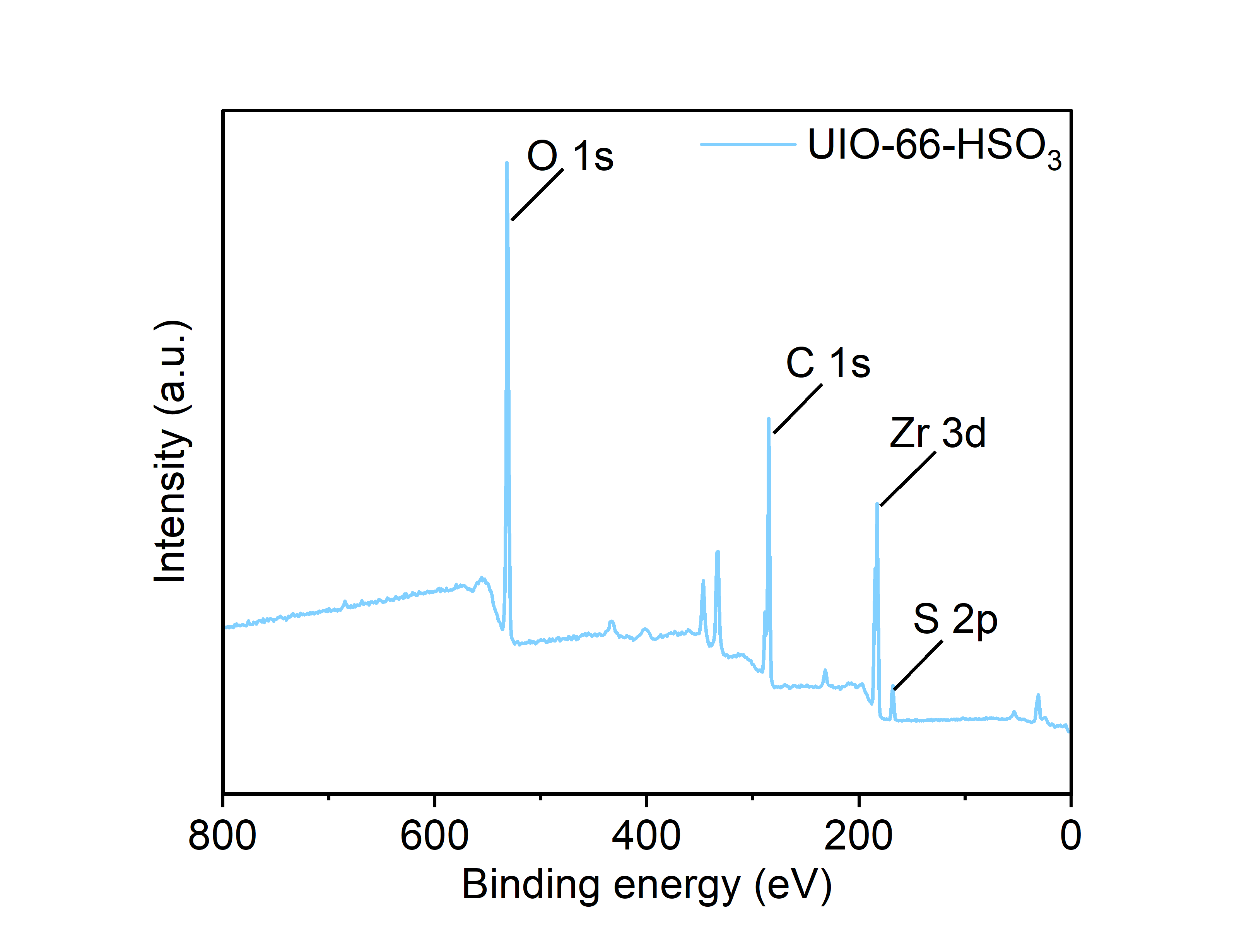

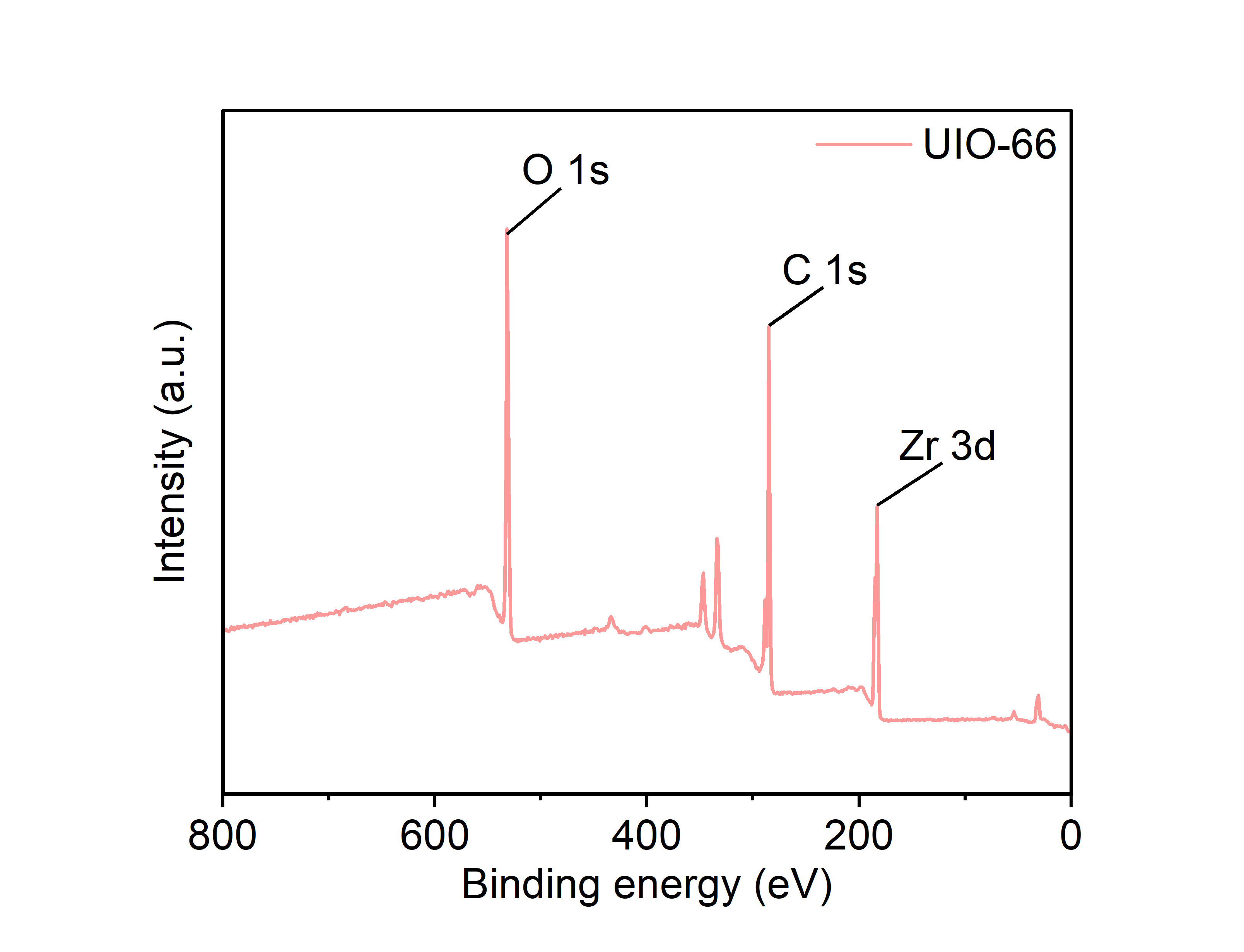

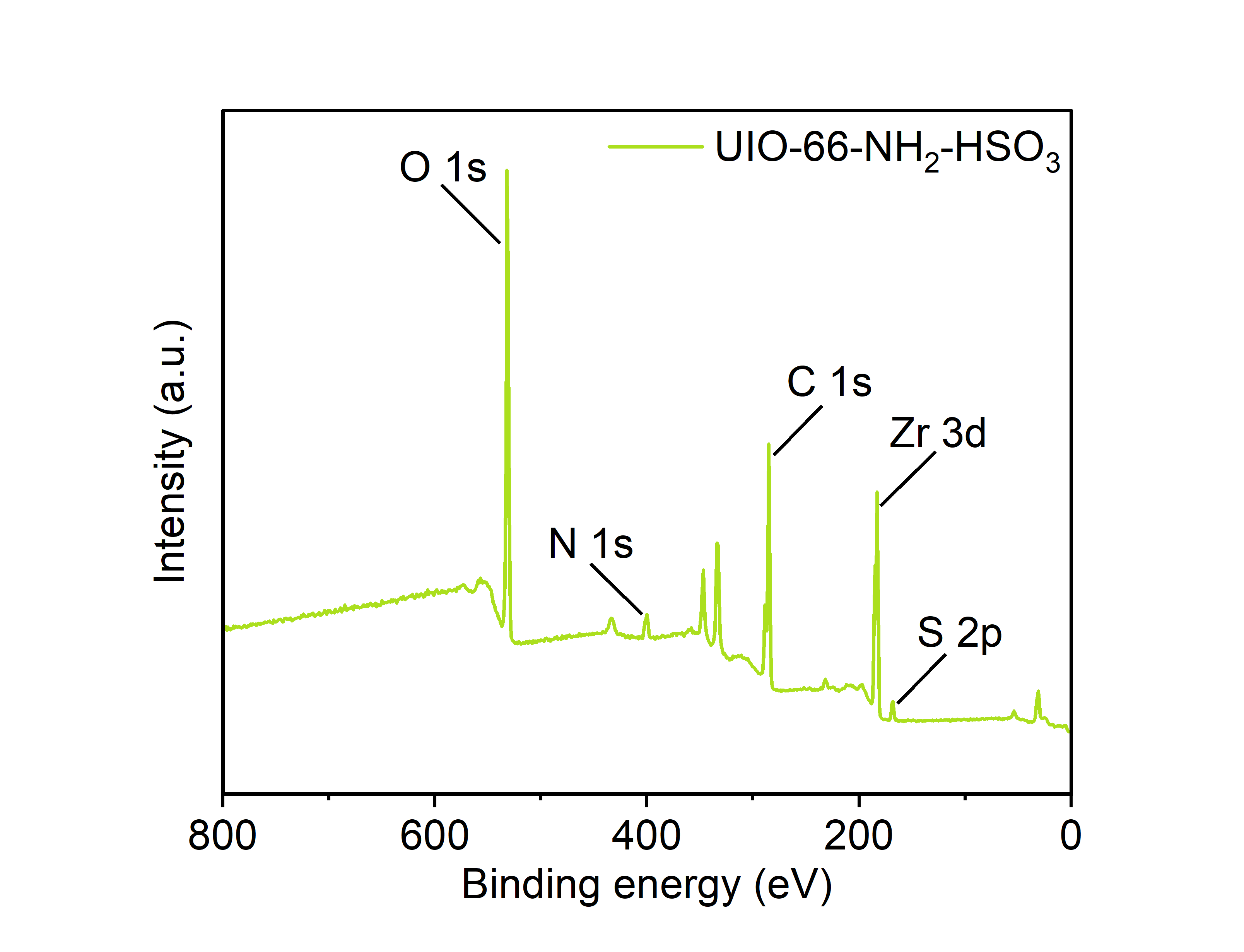

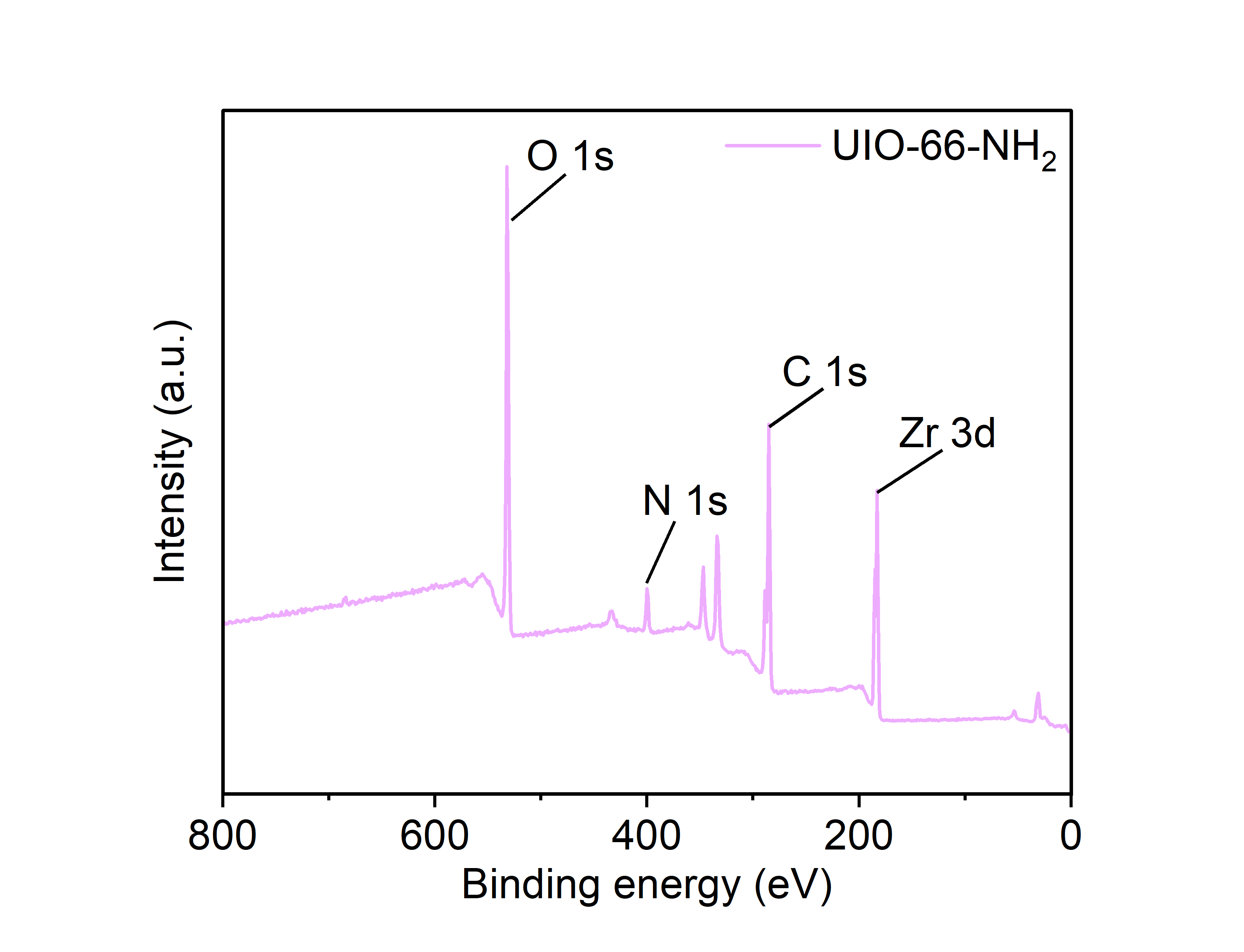
Figure S4.** Full-scan spectra of UIO-66, UIO-66-NH_2_, UIO-66-HSO_3_, and UIO-66-NH_2_-HSO_3_.

# 6. High-resolution XPS spectra of C 1s


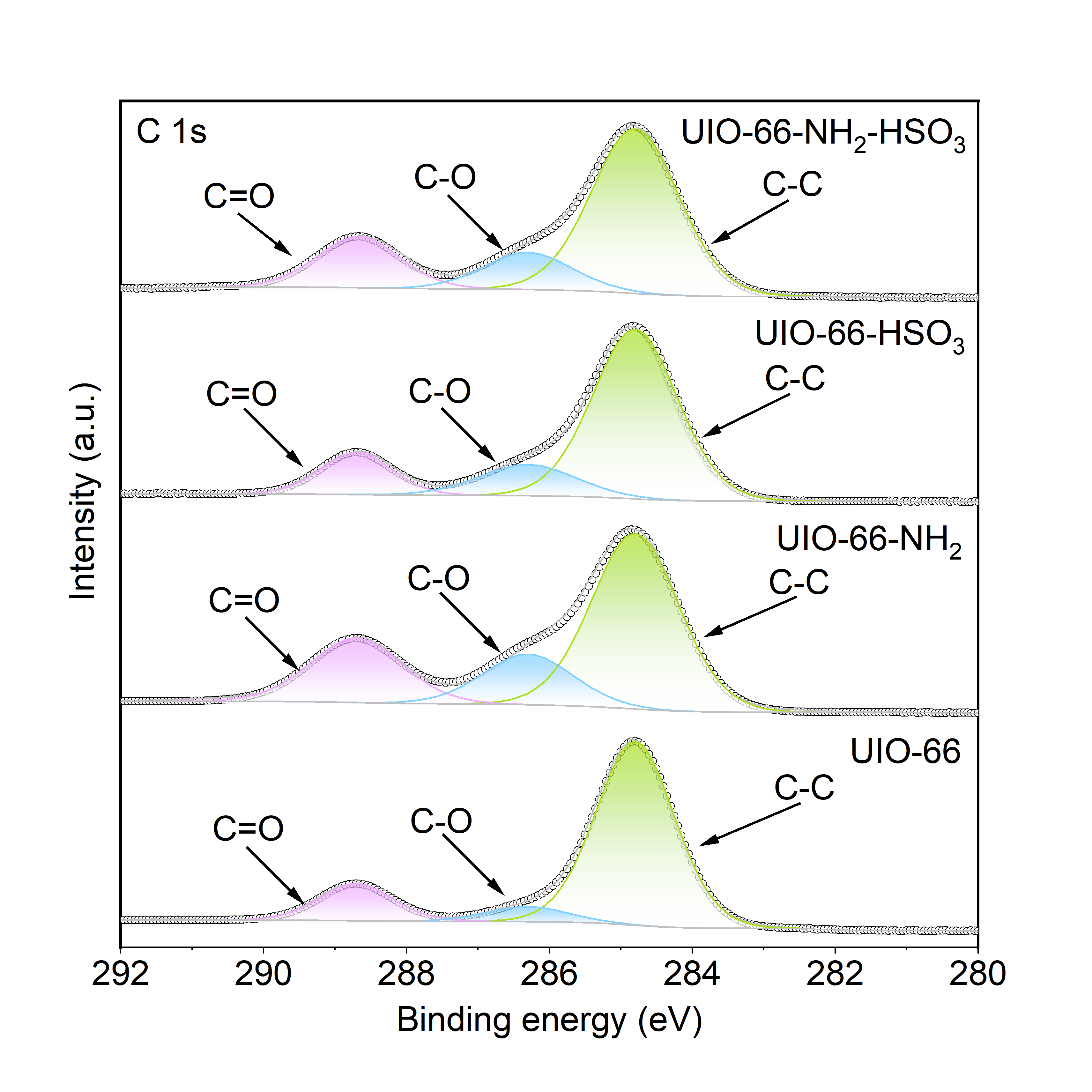
**Figure S5.** High-resolution XPS spectra of C 1s of UIO-66, UIO-66-NH_2_, UIO-66-HSO_3_, and UIO-66-NH_2_-HSO_3_.

# 7. SEM images

**
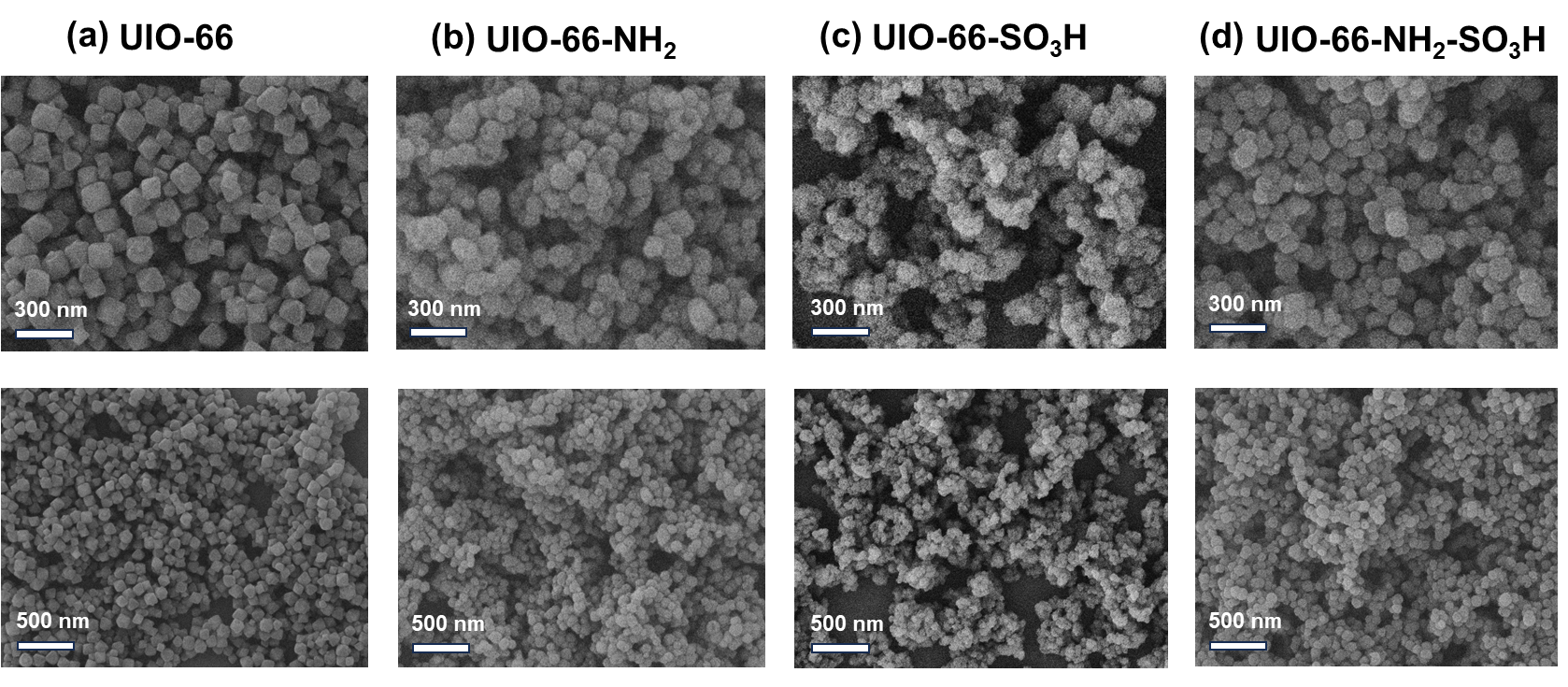
Figure S6.** SEM images of the UIO-66, UIO-66-NH_2_, UIO-66-HSO_3_, and UIO-66-NH_2_-HSO_3_.

# 8. Schematic diagram of mass preparation of modified pp separators


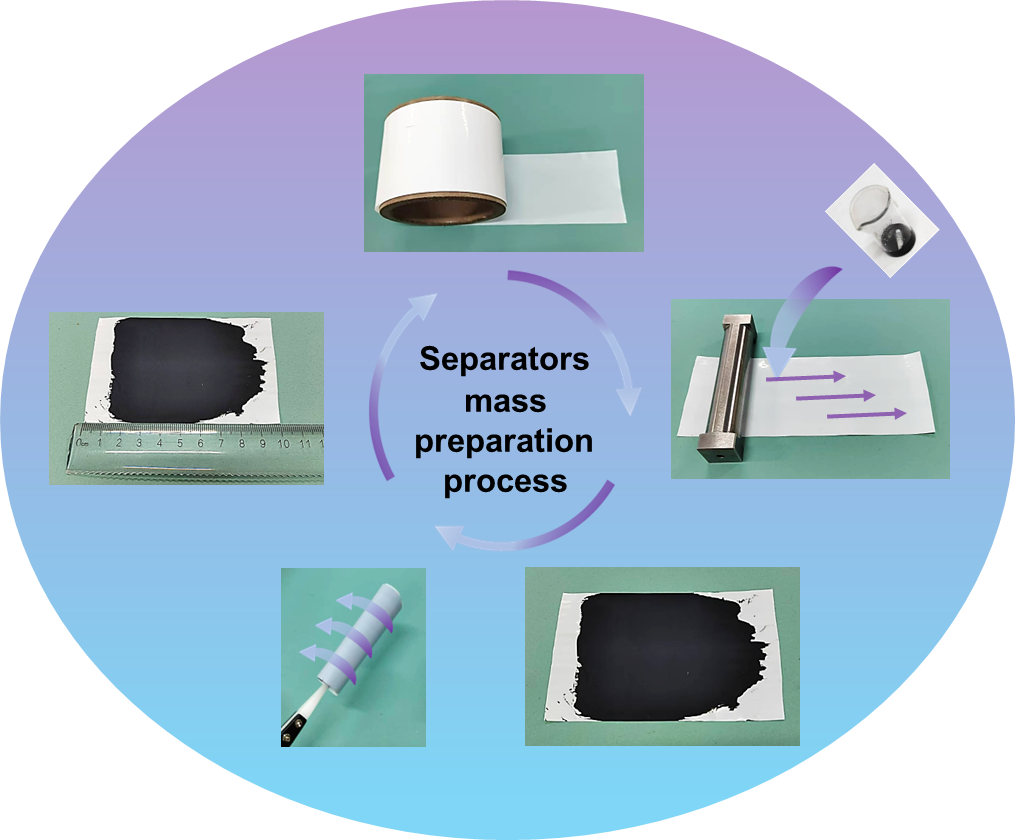


**Figure S7.** Schematic diagram of mass preparation of modified pp separators.

# 9. SEM image of separators surface

**
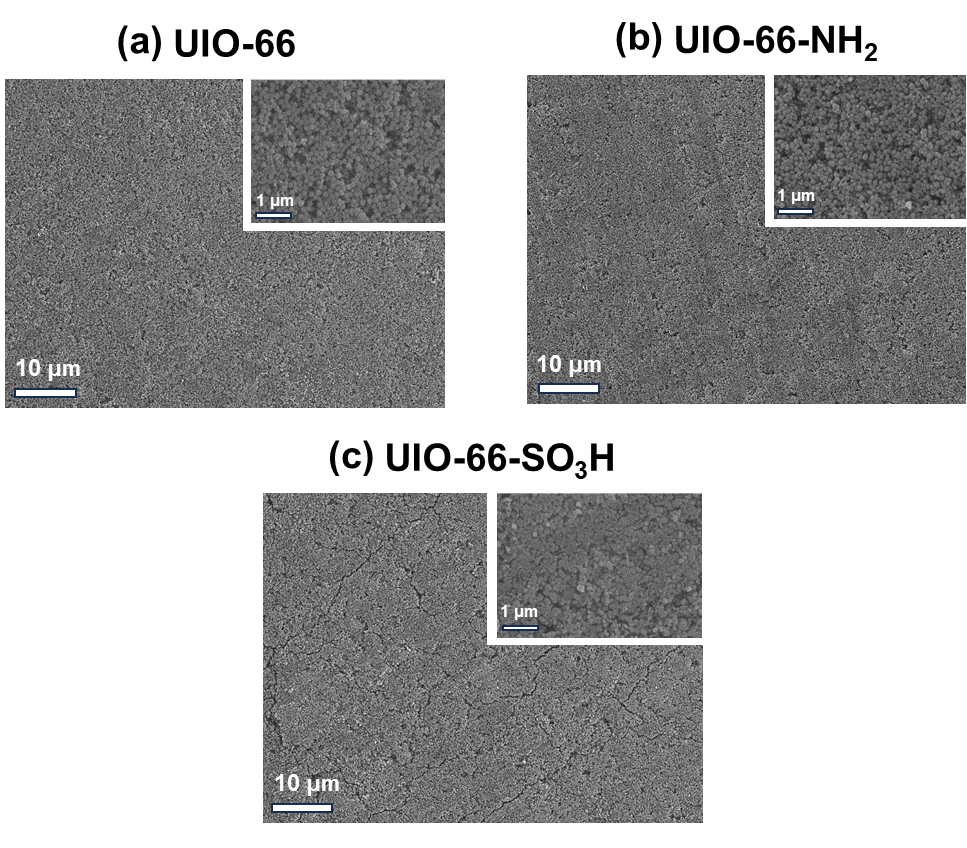
Figure S8.** SEM image of (a) UIO-6 @PP, (b) UIO-66-NH_2_ @PP and (c) UIO-6 -HSO_3_@PP separators surface.

# 10. Photograph of the UIO-66-NH_2_-HSO_3_ modified PP separator


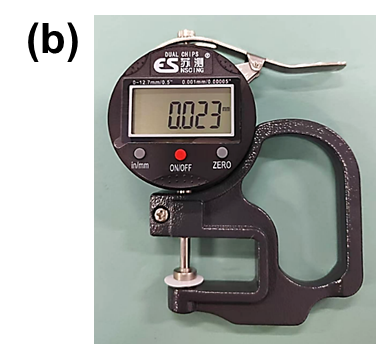

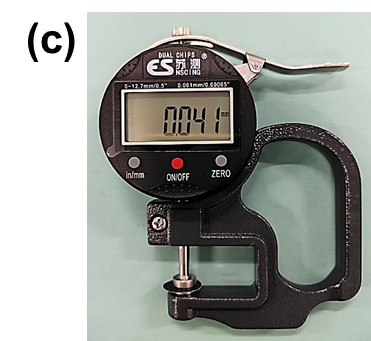

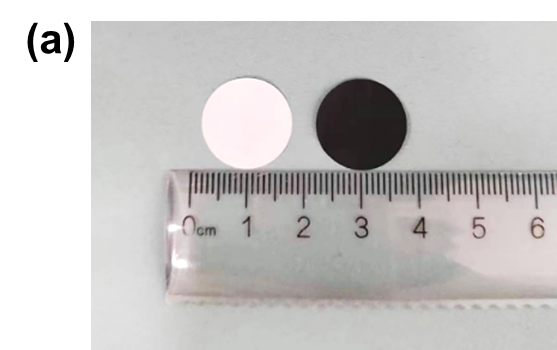


**Figure S9.** (a) Photograph of the UIO-66-NH_2_-HSO_3_ modified PP separator. Thickness gauge measurement results of the (b) pristine PP and (c) UIO-66-NH_2_-HSO_3_@PP separators.

# 11. Optical photographs of Li_2_S_4_ solution permeation tests


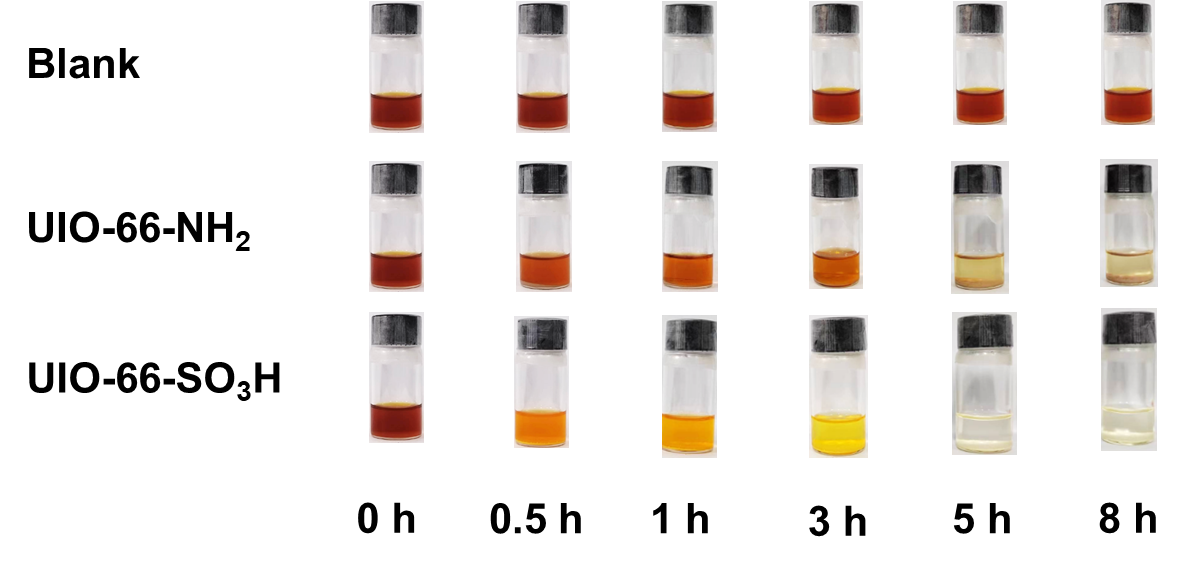


**Figure S10.** Optical photographs of Li_2_S_4_ solution permeation tests of Blank, UIO-66-NH and UIO-66-HSO_3_.

# 12. High-resolution XPS spectra of Zr


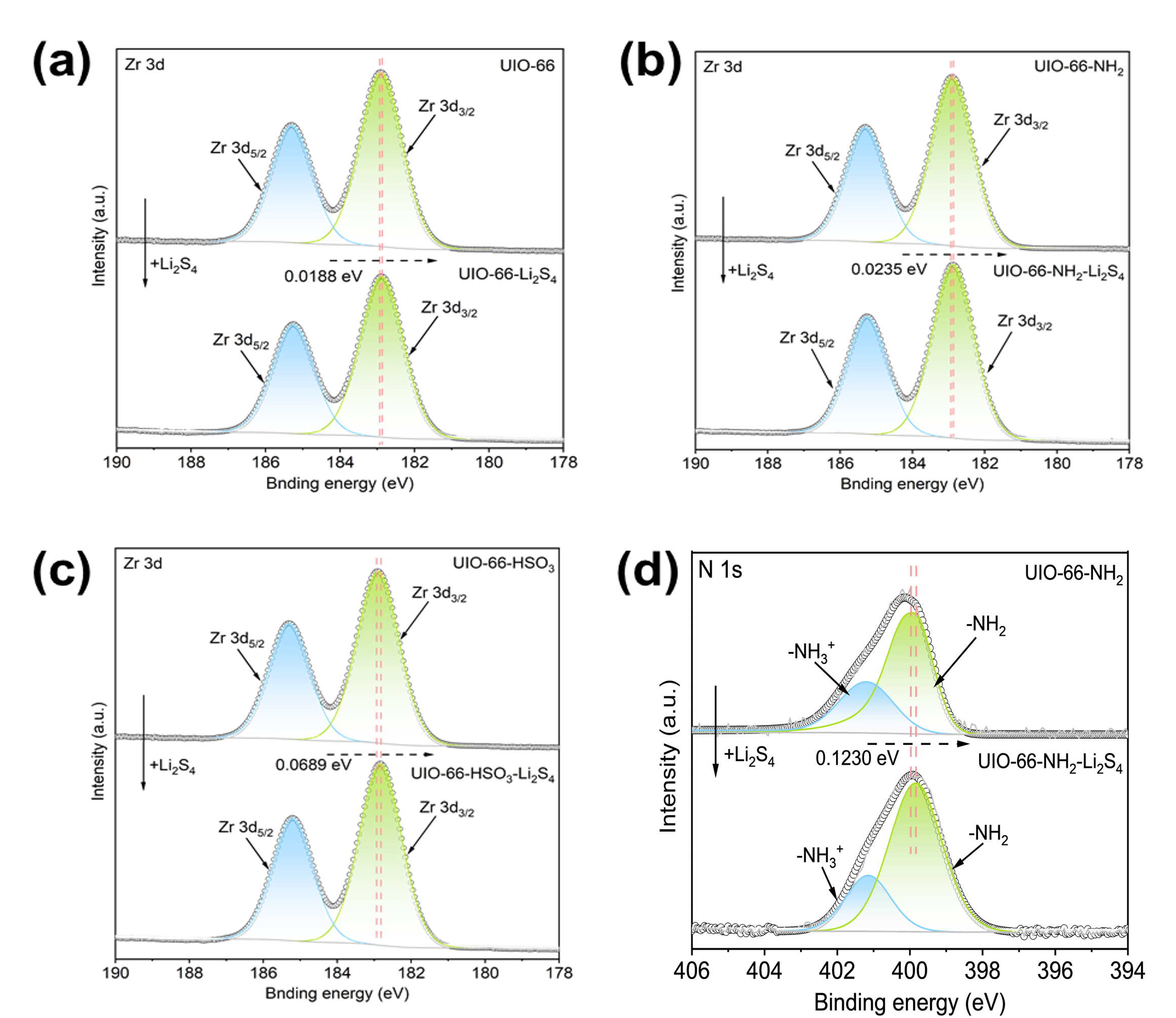


**Figure S11.** High-resolution XPS spectra of Zr 3d in (a) UIO-66 and UIO-66-Li_2_S_4,_ (b) UIO-66-NH_2_ and UIO-66-NH_2_-Li_2_S_4_, and (c) UIO-66-HSO_3_ and UIO-66-HSO_3_-Li_2_S_4_. (d) High-resolution XPS spectra of N 1s in UIO-66-NH_2_ and UIO-66-NH_2_-Li_2_S_4_.

# 13. LiPS diffusion tests in the H-shaped cells


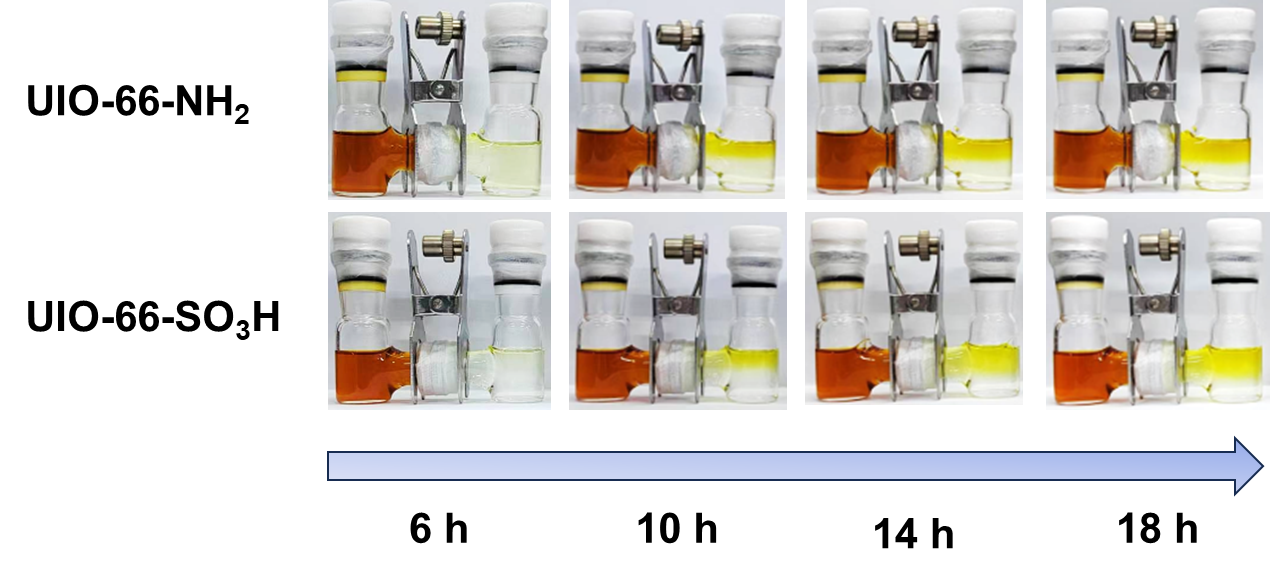
**Figure S12.** LiPS diffusion tests in the H-shaped cells with UIO-66-NH_2_@PP and UIO-66-HSO_3_@PP separators.

# 14. CV curves of the initial third cycles

**
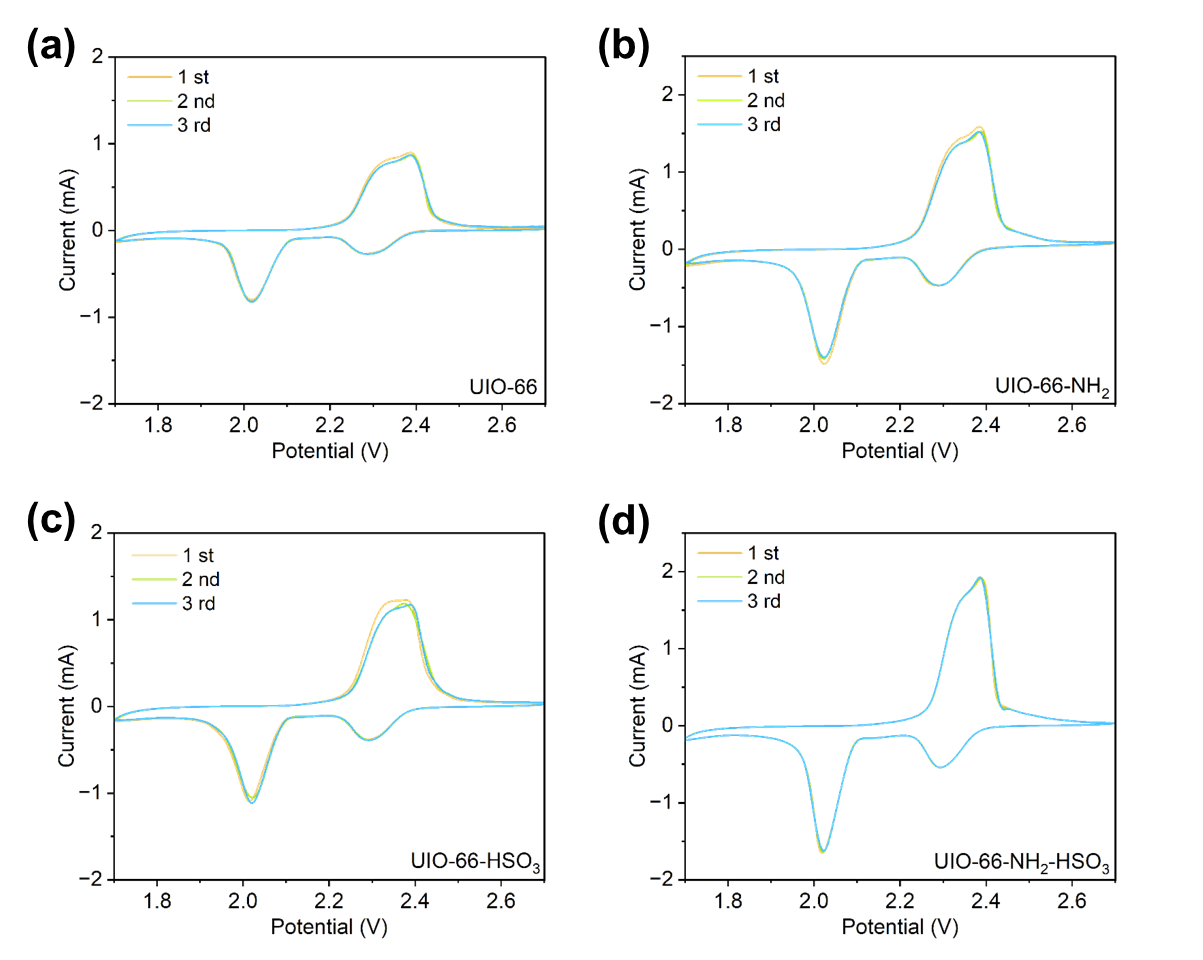
Figure S13.** CV curves of the first third cycles of (a) UIO-66@PP, (b) UIO-66-NH_2_@PP, (c) UIO-66-HSO_3_@PP, and (d) UIO-66-NH_2_-HSO_3_@PP.

# 15. CV curves at various scan rates


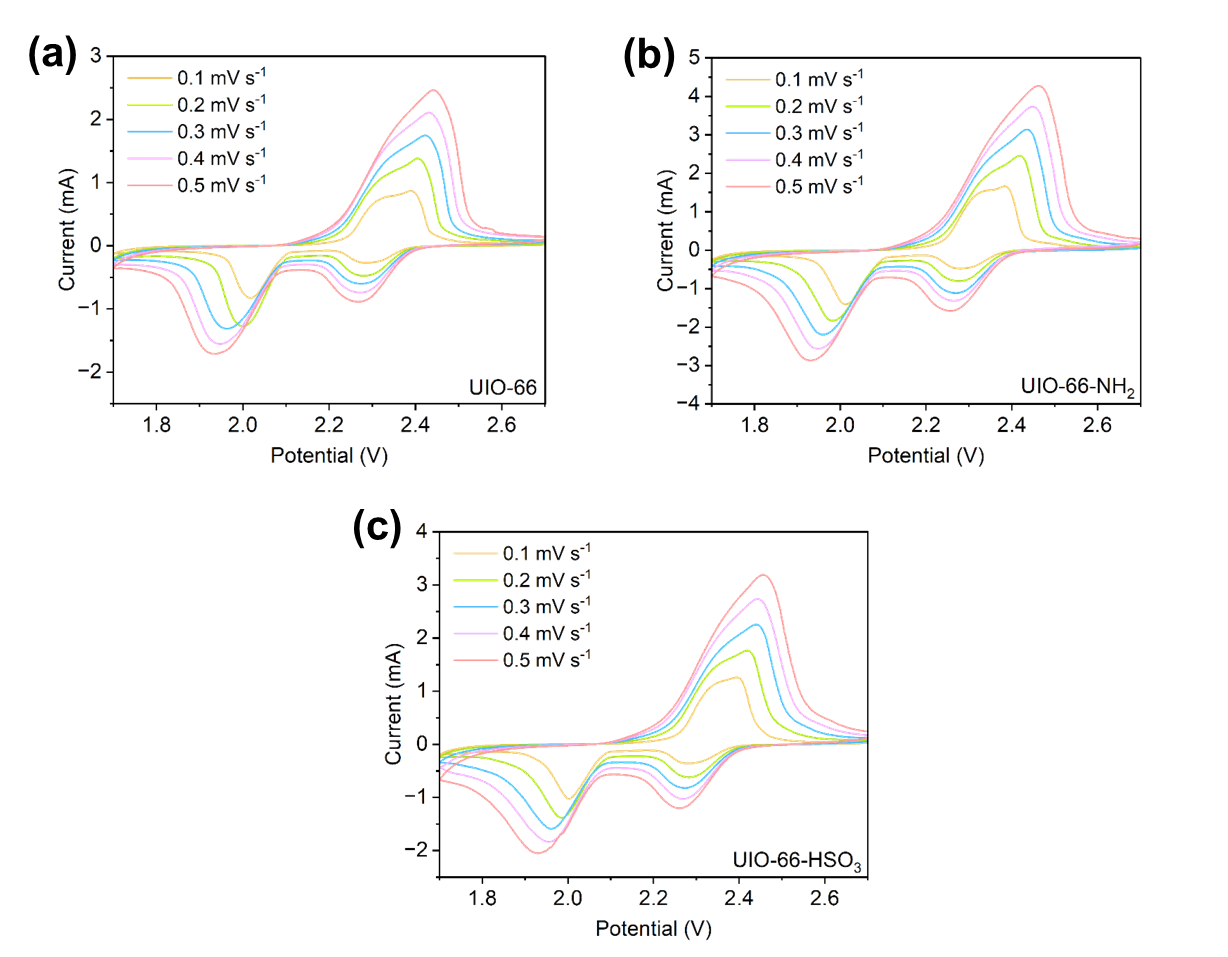
**Figure S14.** CV curves at various scan rates of the (a) UIO-66@PP, (b) UIO-66-NH_2_@PP and (c) UIO-66-HSO_3_@PP.

# 16. GCD profiles


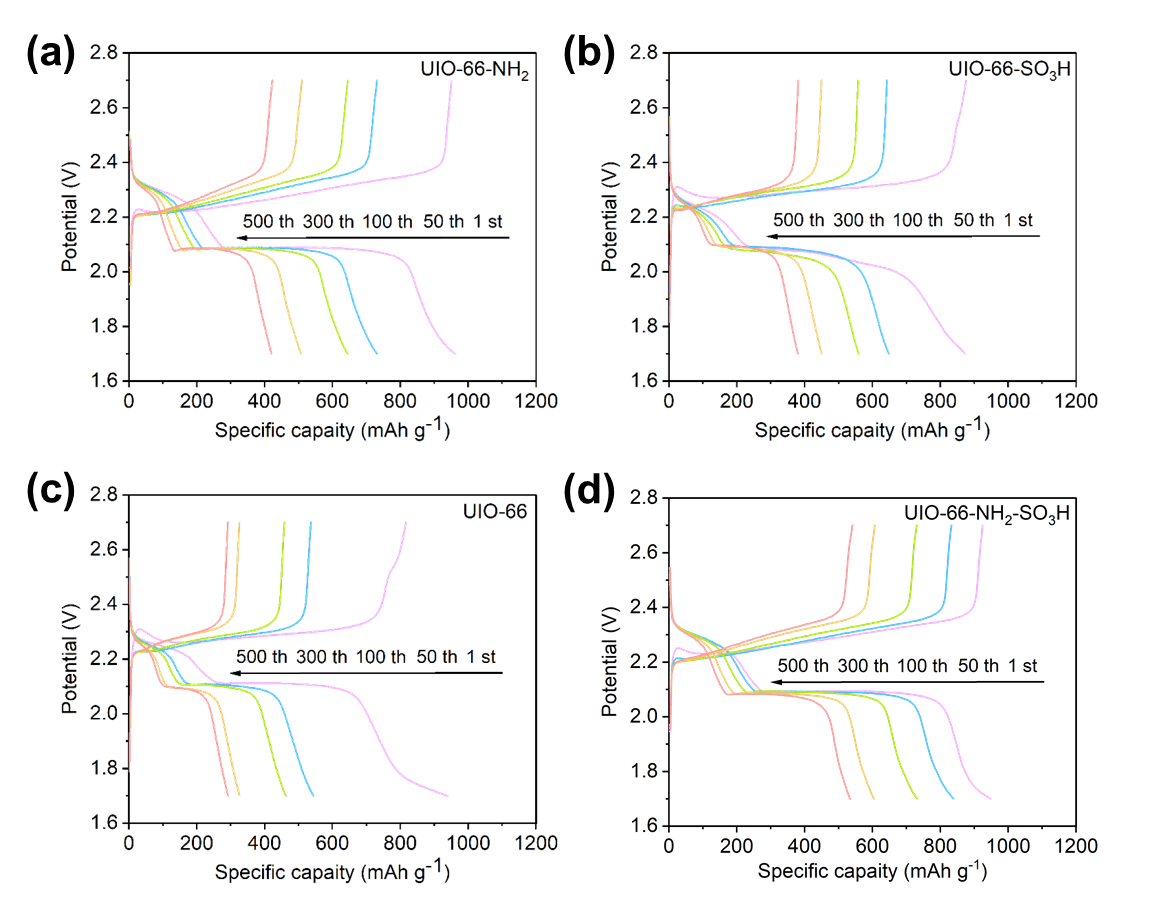
**Figure S15.** GCD profiles of (a) UIO-66@PP, (b) UIO-66-NH_2_@PP, (c) UIO-66-HSO_3_@PP and (d) UIO-66-NH_2_-HSO_3_@PP at 2 C.

# 17. Rate performance of the pristine PP


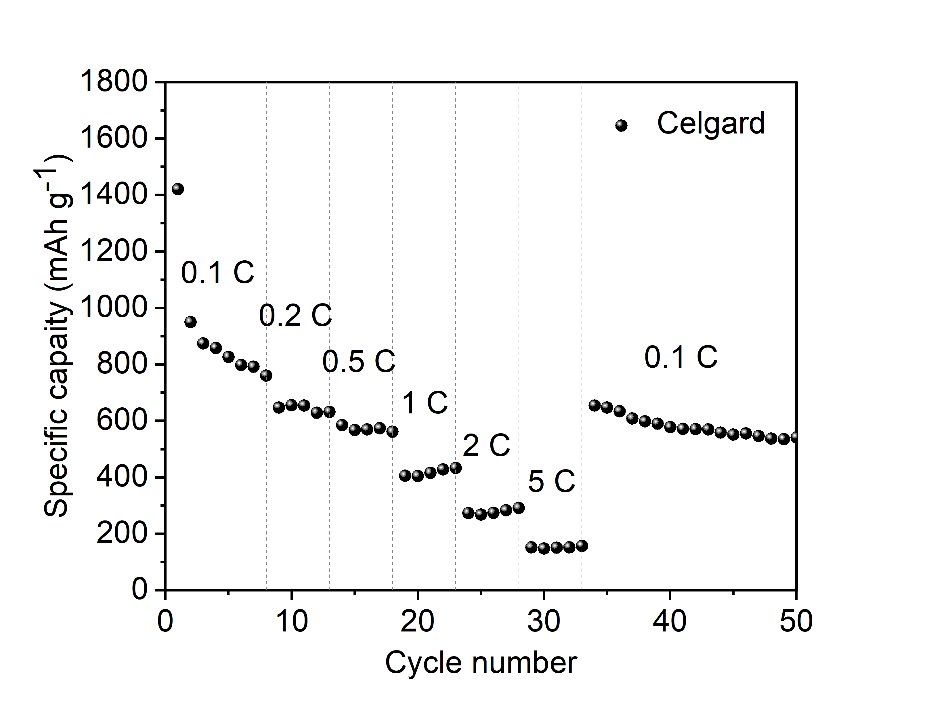
**Figure S16.** Rate performance of the pristine PP at various current densities.

# 18. The charge/discharge profiles


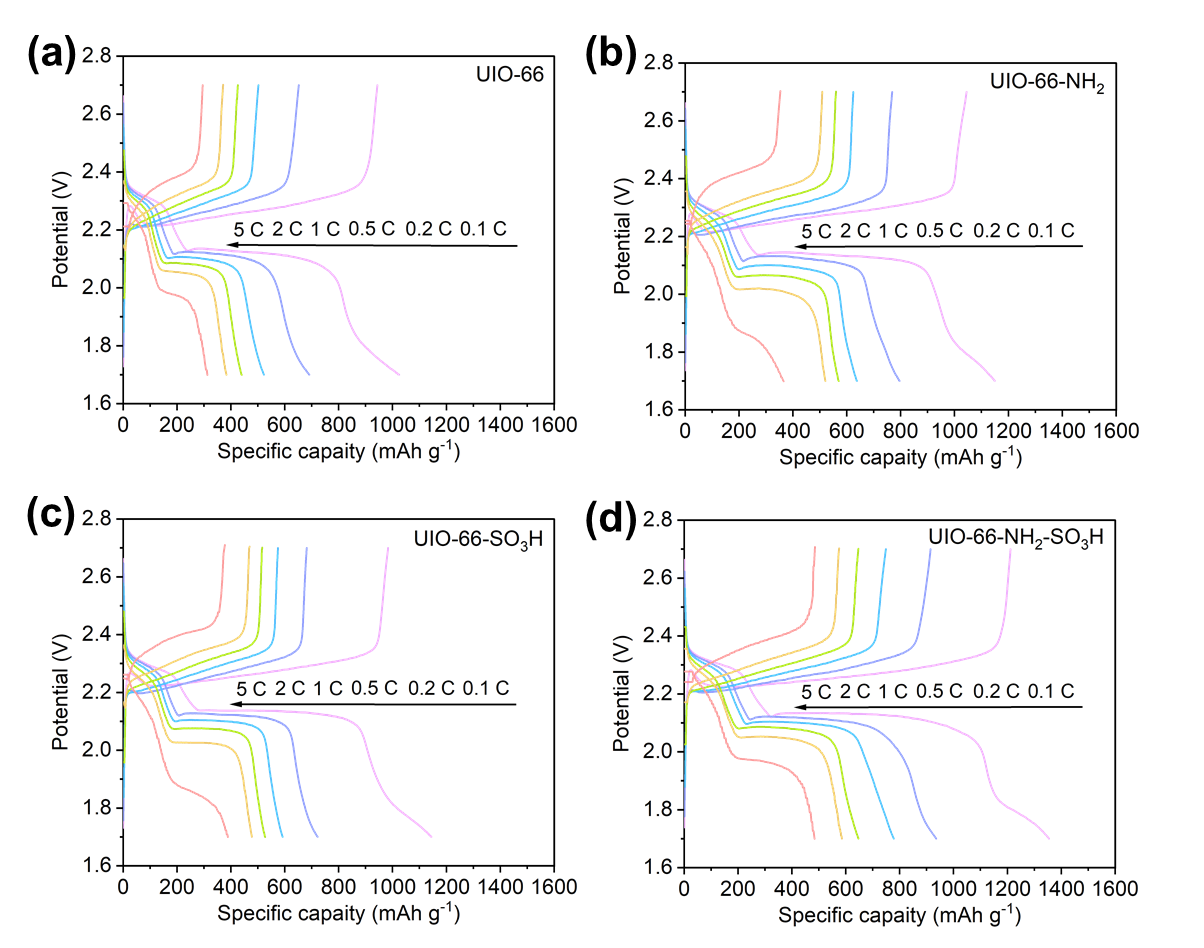
**Figure S17.** The charge/discharge profiles of (a) UIO-66@PP, (b) UIO-66-NH_2_@PP, (c) UIO-66-HSO_3_@PP and (d) UIO-66-NH_2_-HSO_3_@PP at different rates.

# 19. Long-term cycle

**
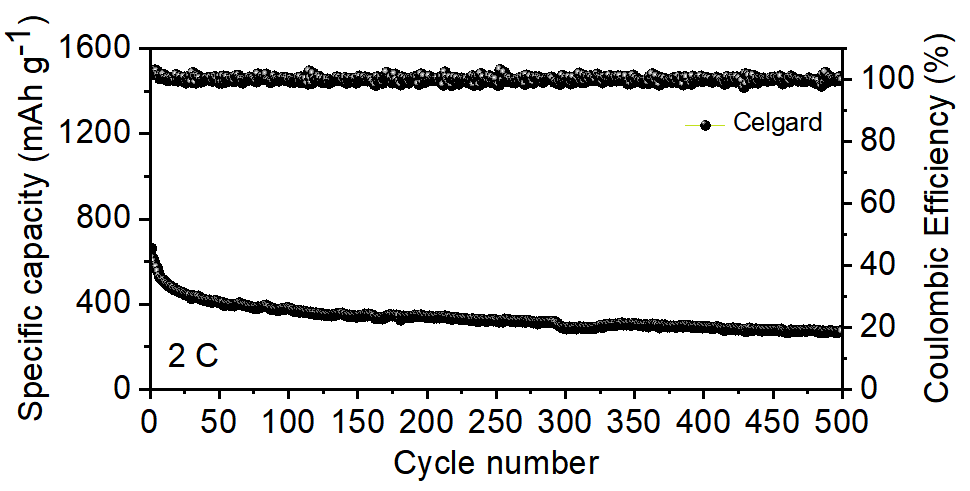
**

**Figure S18.** Long-term cycle performance at 2 C of the pristine PP separators.

# 20. Radar map of the initial and final capacities of all separators

**
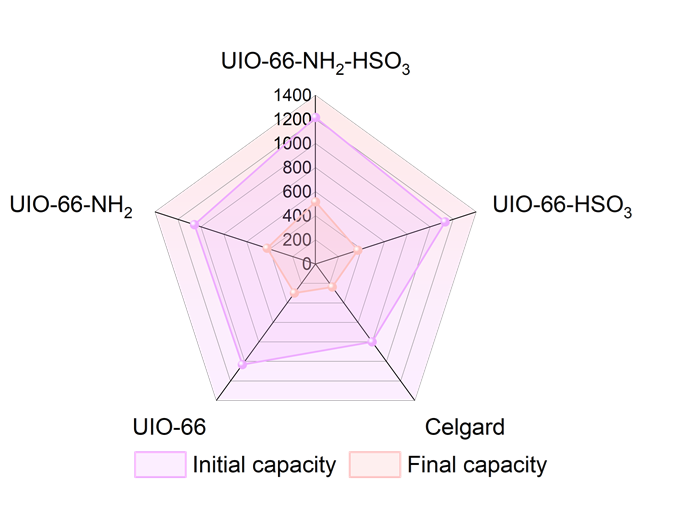
 Figure S19.** Radar map of the initial and final capacities of all separators at 0.5 C.

# 21. Long-term cycle performance at 0.5 C

**
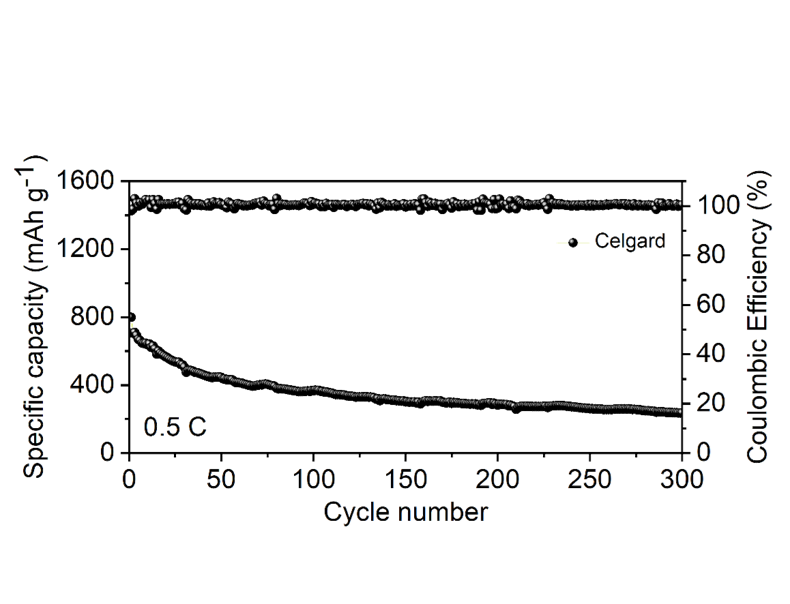
**

**Figure S20.** Long-term cycle performance at 0.5 C of the pristine PP separators.

# 22. GCD profiles

**
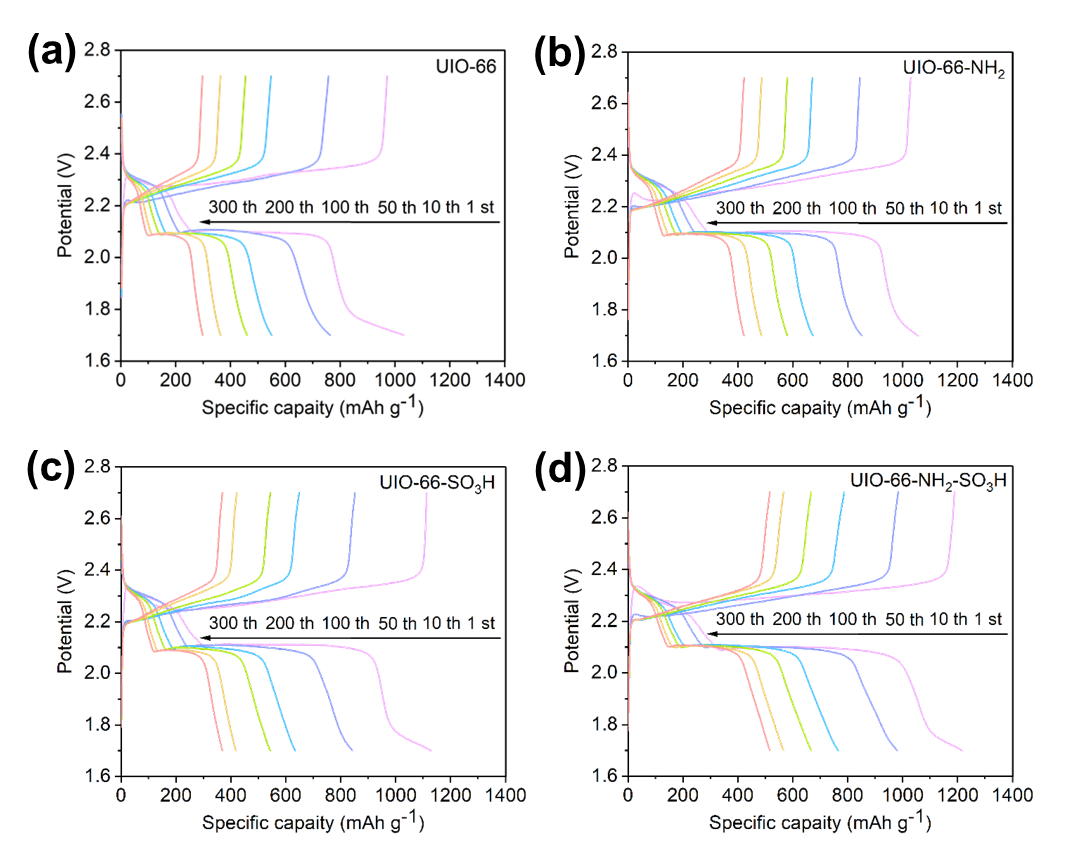
**

**Figure S21.** GCD profiles of (a) UIO-66@PP, (b) UIO-66-NH_2_@PP, (c) UIO-66-HSO_3_@PP and (d) UIO-66-NH_2_-HSO_3_@PP at 0.5 C.

# 23. Contour maps of in situ UV-vis spectra

**
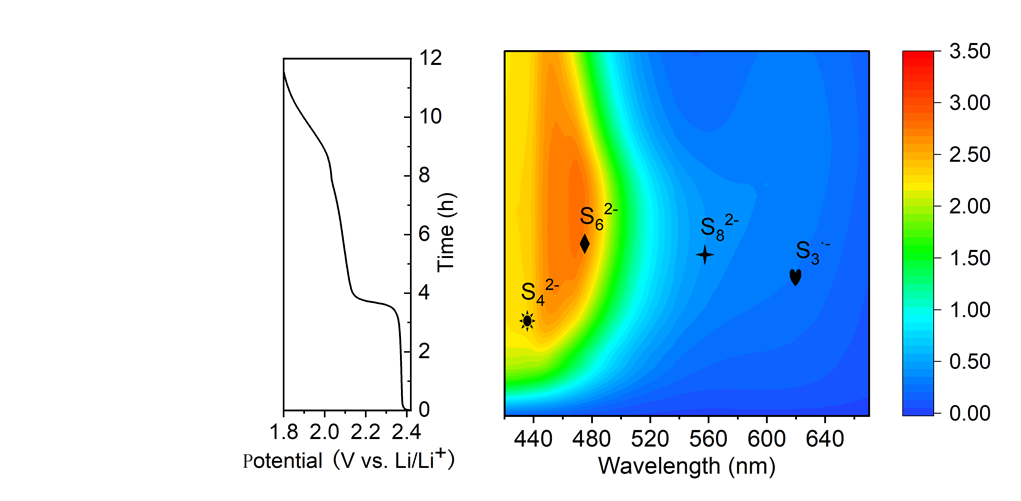
**

**Figure S22.** Contour maps of in situ UV-vis spectra and the corresponding discharge profile of the pristine PP separator.

# 24. In-situ electrochemical impedance spectroscopy DRT analysis results of unmodified separator


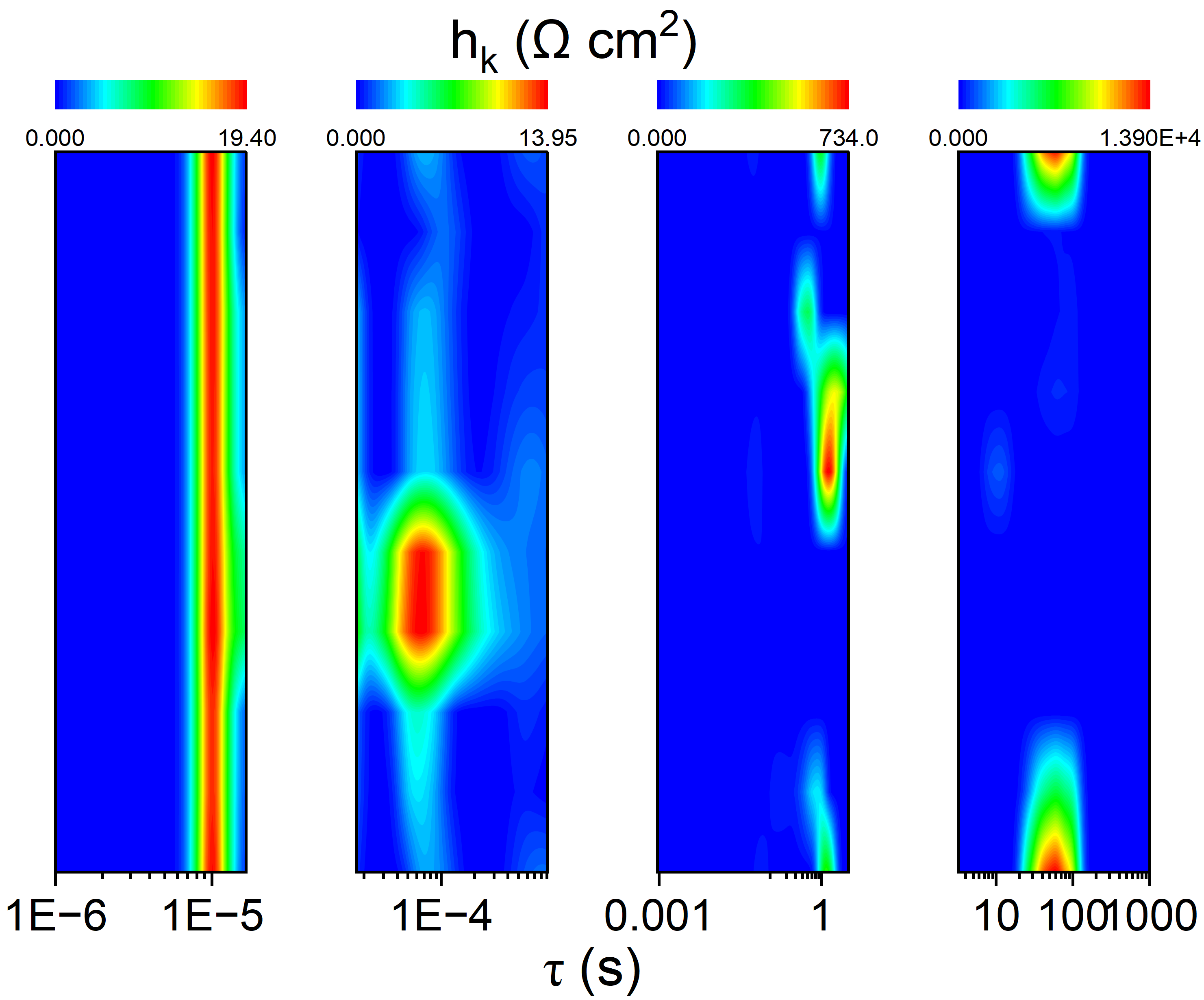


**Figure S23.** In-situ electrochemical impedance spectroscopy DRT analysis results of unmodified separator.

# 24. The optimized configurations for interaction energies of S_4_^2−^


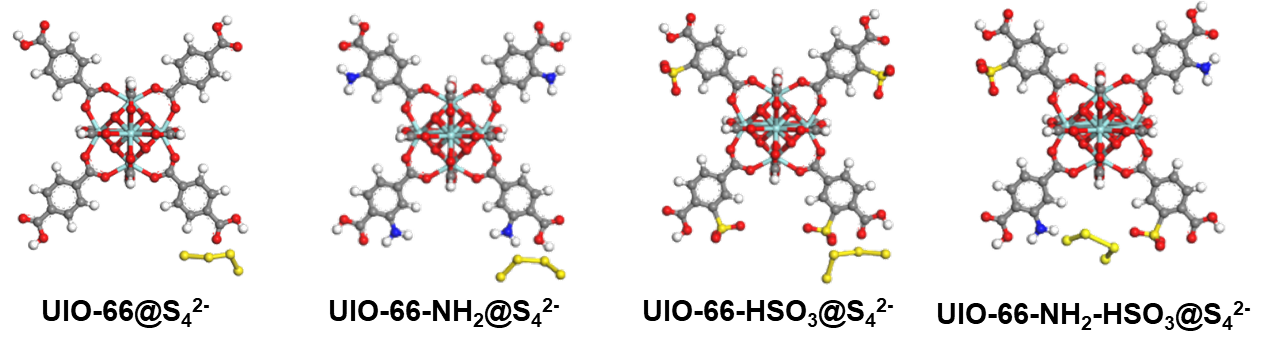


**Figure S24.** The optimized configurations for interaction energies of S_4_^2−^ with UIO-66, UIO-66-NH_2_, UIO-66-HSO_3_, and UIO-66-NH_2_-HSO_3_.

# 25. The optimized configurations for adsorption energy of Li_2_S_4_ molecules


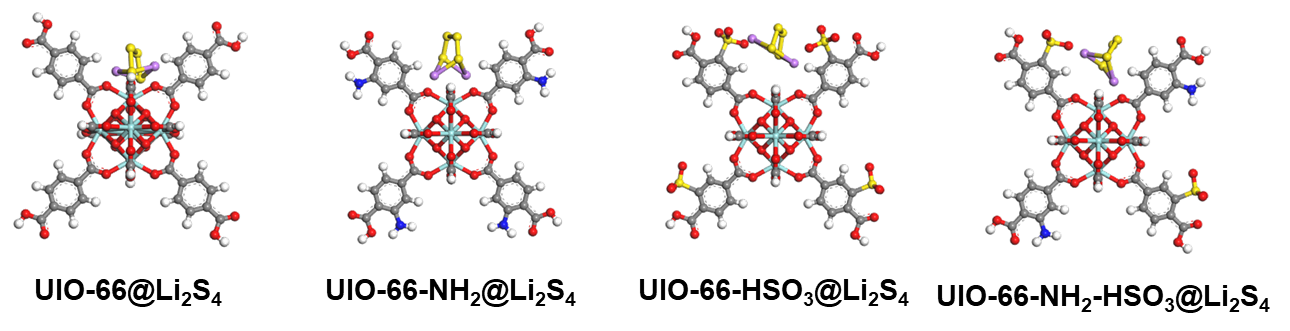


**Figure S25.** The optimized configurations for adsorption energy of Li_2_S_4_ molecules with UIO-66, UIO-66-NH_2_, UIO-66-HSO_3_, and UIO-66-NH_2_-HSO_3_.

# 26. The optimized configurations for binding energy of Li^+^


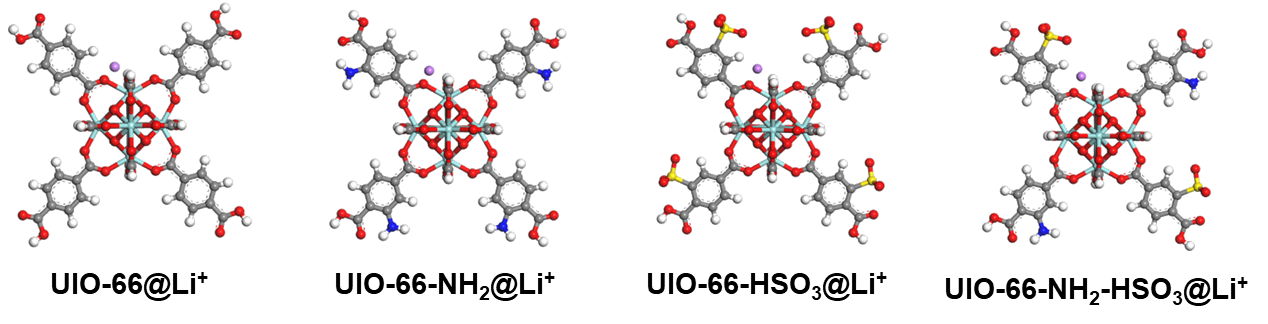


**Figure S26.** The optimized configurations for binding energy of Li^+^ with UIO-66, UIO-66-NH_2_, UIO-66-HSO_3_, and UIO-66-NH_2_-HSO_3_.

s
